# Metabolic and Network Pharmacological Analyses of the Therapeutic Effect of *Grona styracifolia* on Calcium Oxalate-Induced Renal Injury

**DOI:** 10.3389/fphar.2021.652989

**Published:** 2021-06-25

**Authors:** Wei Chen, Yachen Si, Jin Cheng, Jiarong Ding, Hongxia Zhao, Wenrui Liu, Qishan Lin, Jiebin Hou, Zhiyong Guo

**Affiliations:** ^1^ Department of Nephrology, Shanghai Changhai Hospital, Navy Medical University, Shanghai, China; ^2^ School of Pharmacy, Navy Medical University, Shanghai, China; ^3^ RNA Epitranscriptomics and Proteomics Resource, Department of Chemistry, University at Albany, Albany, NY, United States; ^4^ Department of Nephrology, The Second Medical Centre (National Clinical Research Center for Geriatric Diseases), Chinese PLA General Hospital, Beijing, China

**Keywords:** traditional medicine, biological network, metabolomics, integrative pharmacology, renal injury

## Abstract

*Grona styracifolia* (Osbeck) Merr. (GS), a popular folk medicine, is clinically applied to treat nephrolithiasis. In this study, a urinary metabolic analysis was performed in a mouse model of renal calcium oxalate (CaOx) crystal deposition to identify the differentially altered metabolites in mice with oxalate-induced renal injury and explore the therapeutic mechanisms of GS against nephrolithiasis. Twenty-four mice were randomly divided into the control, oxalate and GS-treated groups. A metabolomics approach based on ultra-high-performance liquid chromatography coupled with quadrupole-time-of-flight mass spectrometry (UHPLC-Q-TOF/MS) was used to analyze the metabolic profiles of the urine samples. In addition, network pharmacology analysis was performed with different databases. As a result, the protective effects of GS were verified by measuring biochemical parameters and detecting crystal deposition. Fifteen metabolites were identified as the differentially altered metabolites in mice with crystal-induced renal injury. Most were involved in amino acid and fatty acid metabolism. Thirteen of these metabolites showed a reversal trend following GS treatment. A component-target-metabolite network was further constructed and nine overlapping target proteins of GS and the differentially altered metabolites were discovered. Among these proteins, the expression of estrogen receptor 2 (ESR2) in renal tissues was significantly down-regulated while androgen receptor (AR) expression was obviously increased in the oxalate group compared with the control group. These changes were reversed by the GS treatment. In conclusion, GS exerts its therapeutic effect by regulating multiple metabolic pathways and the expression of ESR and AR in mice with oxalate-induced renal injury.

## Introduction

Kidney stone disease, also known as nephrolithiasis, is a common disease jeopardizing the health of 7–13% of the population worldwide (Sorokin et al., 2017). Nephrolithiasis is considered a systemic disease because of its significant association with chronic kidney disease, cardiovascular events and metabolic syndrome (including type 2 diabetes, obesity, hypertension and dyslipidemia), as reported by numerous epidemiological studies (Sakhaee, 2008). Calcium oxalate (CaOx) is the most common inorganic matrix found in kidney stones and crystals. CaOx crystals, an early form of kidney stones, have also been shown to cause apoptosis in renal tubular cells (Mulay et al., 2013). The supersaturation of the urine is the first step in the formation of kidney crystals, followed by the progression of nucleation, growth and aggregation (Kolbach-Mandel et al., 2015). Renal injury induced by kidney stones has been observed throughout the process of stone formation. Metabolic disturbances are also promoted by crystal-induced renal injury. Thus, metabolic disturbances resulting from renal injury during crystal formation should be addressed.

*Grona styracifolia* (Osbeck) H. Ohashi and; K. Ohashi (GS), a popular traditional medicine (also known as “Guang Jin Qian Cao” in Chinese), is widely distributed in southern China and is clinically applied to treat nephrolithiasis. GS has been shown in a previous study to alleviate CaOx deposition in a murine model and has been reported to effectively relieve the apoptosis of tubular cells and prevent the oxidative stress changes induced by crystals (Zhou et al., 2018). However, no studies have been performed to evaluate the effects of GS on nephrolithiasis from the perspective of metabolism.

Metabolomics is a scientific study of the systemic metabolic changes in complex living systems in response to pathophysiological stimuli and has been effectively applied to reveal metabolic changes in diverse types of renal injury (Fan et al., 2018). In addition, the concept of network pharmacology has been developed due to the rapid development of bioinformatics. Network pharmacology is more effective for establishing a “compound-protein/gene-disease” network and revealing the regulatory effects of small molecules in a high-throughput manner. This approach is very powerful for the analysis of drug combinations, especially traditional medicinal herbs (Zhang et al., 2019). A combination of network pharmacology and metabolomics can establish the relationships of herb targets and endogenous metabolic responses to further reveal the molecular mechanisms of the herb based on a component-target-metabolite network. In the present study, a urinary metabolomics-based approach integrated with network pharmacology was conducted to investigate the potential effects of GS on a mouse model of crystalline nephropathy characterized by renal CaOx deposition.

## Materials and Methods

### Preparation and Quality Control of GS

*Grona styracifolia* (Osbeck) H. Ohashi and K. Ohashi (GS) was purchased from Anguo (Hebei, China) and was authenticated by Prof. Lianna Sun (School of Pharmacy, Navy Medical University, Shanghai, China). The purchased herbs were extracted using standard methods according to the Chinese Pharmacopoeia (Yu et al., 2015). First, the entire GS plant was ground into a powder and was filtered through a 250 μm mesh. Next, 200 g of the powder was extracted with 1,000 ml of 80% methanol for 2 h using a ultrasonicator. The extract solution was filtered and then concentrated using a rotary evaporator, followed by drying to a powder under a vacuum at 60°C. The GS was parallel-extracted six times and the average yield of the methanol extract was 13.1% (w/w).

The ultra-high-performance liquid chromatography coupled with quadrupole-time-of-flight mass spectrometry (UHPLC-Q-TOF/MS) approach was used to test the reproducibility of GS extraction. Based on previous research and the literature (Zhou et al., 2012; Yu et al., 2015), four major constituents of GS, including schaftoside, isovitexin, luteolin, and apigenin, were selected for further analysis. The relative standard derivation (RSD) values of the peak areas of each component of the product were calculated in six parallel extracts of GS. The standard substances (NatureStandard, Shanghai, China) of these four constituents were also analyzed for comparison. A detailed description of the methodology is provided in the Supplemental Materials section (Supplementary Methods S1).

### Cell Model and Treatment

The human proximal tubular cell line, HK-2 cells were obtained from the ATCC (Manassas, United States). Cells were incubated with sodium oxalate (NaOx) at 1 mM for 12 h to establish a cell model. Potassium citrate (K3Cit) is known for preventing the formation of kidney stone and was used as a positive control. HK-2 cells were divided into five groups (six wells per group): control groups (no treatment), model group (NaOx), K3Cit group (NaOx +1.0 mg/ml K3Cit), 25 μg/ml GS group (NaOx +25 μg/ml GS), 50 μg/ml GS group (NaOx +50 μg/ml GS) and 100 μg/ml GS group (NaOx +100 μg/ml GS). The levels of IL-6 and TNF-α in the cell supernatant were detected using ELISA kits (MultiSciences, Hangzhou, China). Simultaneously, lactate dehydrogenase (LDH) release was determined according to the instructions provided with the kits (Jiancheng Bioengineering, Nanjing, China).

### Animal Experiment and Sample Collection

This experiment was approved by the Animal Ethics Committee of Navy Medical University (license: SCXK (Hu) 2017-0004) and was performed in accordance with the Guiding Principles for the Care and Use of Laboratory Animals. Twenty-four wild-type male C57B/L6 mice aged seven to eight weeks were purchased from the Shanghai SLAC Laboratory Animal Co., Ltd. (production license: SCXK (Hu) 2017-0005), provided with standard food and water, and housed at a temperature of 20–25°C and relative humidity of 55–65%. After one week, the 24 mice were randomly divided into four groups: the control group, oxalate group, oxalate + low dose GS group and oxalate + high dose GS group (*n* = 6).

The mice were intraperitoneally injected with 100 mg/kg glyoxylate once daily for seven days to establish the oxalate-induced renal injury model (*n* = 18), while the mice in the control group received an equal volume of saline. Two days after the first injection of glyoxylate, the mice received a daily gastric perfusion of the GS extract at a low dose of 10 ml/kg (equivalent to 500 mg/kg body weight of the whole plant powder, *n* = 6) and a high dose of 2 0 ml/kg (equivalent to 1,000 mg/kg body weight of the whole plant powder, *n* = 6) for five consecutive days, while the mice in the control and oxalate groups received an equal volume of saline. After the last gavages, all the mice were placed in metabolic cages for 24 h to collect urine samples, which were stored at −80°C until the biochemical and metabolic analysis. Then, the mice were all anesthetized with sodium thiopental. Blood samples were collected and separated by centrifugation at 3,000 rpm for 10 min and stored at −80°C until the biochemical analysis. After *in situ* cardiac perfusion, the right kidneys were immediately removed and stored at −80°C until further biochemical analysis and western blot. Next, the left kidneys were removed and fixed with 10% buffered formalin for pathological analysis.

### Biochemical Analysis and Detection of Crystal Deposition

Serum creatinine (SCr) and blood urea nitrogen (BUN) levels were detected using creatinine assay and urea assay kits, respectively (Jiancheng Bioengineering, Nanjing, Jiangsu, China). The urine calcium and creatinine levels were measured using kits (Jiancheng Bioengineering, Nanjing, Jiangsu, China) and the ratio of urine calcium to creatinine (UCa/Cr) was calculated. The renal tissues were placed in prechilled normal saline and phosphate-buffered saline (PBS, pH = 7.4) to produce 10% kidney homogenates. Then, calcium contents in renal tissues were determined with kits (Jiancheng Bioengineering, Nanjing, Jiangsu, China).

The kidney samples were embedded in paraffin and sectioned at a thickness of 3 μm. The deposition of CaOx crystals in representative sections was identified using a polarizing microscope.

### Reverse Transcription-Quantitative Polymerase Chain Reaction

Total RNA was extracted from the renal tissues using TRIzol reagent (Invitrogen, Carlsbad, CA, United States) according to the manufacturer’s instructions. The RNA concentration and purity were determined by measuring the absorbance at 260/280 nm. Reverse transcription kits (Vazyme, Nanjing, Jiangsu, China) were used to reverse transcribe RNA into cDNAs. The expression of kidney injury molecule 1 (KIM-1; forward: 5′-ATG​AAT​CA-GAT​TCA​AGT​CTT​C-3′ and reverse: 5′-TCT​GGT​TTG​TGA​GTC​CAT​GTG-3′) was determined using the SYBR Green PCR kit (Yeasen, Shanghai, China).

### UHPLC-Q-TOF/MS Analysis of Urine Samples

The UHPLC-Q-TOF/MS analysis was performed on an Agilent 1,290 Infinity LC system equipped with an Agilent 6,538 Accurate Mass Quadrupole Time-of-Flight mass spectrometer (Agilent Technologies, Santa Clara, CA, United States). Chromatographic separations were performed at 40°C on an ACQUITY UPLC HSS T3 column (2.1 mm × 100 mm, 1.8 μm, Waters, Milford, MA, United States). The mobile phase consisted of 0.1% formic acid (A) and ACN modified with 0.1% formic acid (B). The optimized UPLC elution conditions were as follows: 0–1 min, 2% B; 1–6 min, 2–20% B; 6–9 min, 20–50% B, 9–13 min, 50–95% B; and 13–15 min, 95% B. The post time was set to 5 min for system equilibration. The flow rate was set to 0.35 ml/min, and the injection volume was 4 μL. The auto-sampler was maintained at 4°C.

An electrospray ionization source (ESI) was used both in positive and negative mode. The optimized conditions were as follows: capillary voltage, 4 kV in the positive mode and 3.5 kV in the negative mode; drying gas flow, 11 L/min; gas temperature, 350°C; nebulizer pressure, 45 psig; fragmentor voltage, 120 V; and skimmer voltage, 60 V. Data were collected in the profile mode from 50 to 1,100 m/z. Potential metabolites were further analyzed using MS/MS, and the collision energy ranged from 10 to 40 eV.

### Data Processing and Statistical Analysis

The raw UPLC data were converted into a common data format (mzData files) using Agilent Mass Hunter Qualitative software, in which the isotope interferences were excluded with a threshold set to 0.1%. The XCMS program (http://metlin.scripps.edu/download/) was used for peak detection, retention time (RT) alignment and peak integration to generate a visual data matrix. After filtering the ions based on an 80% rule, the data from each sample were normalized to the total intensity to correct for the MS response shift. Then, the three-dimensional data, including RT-m/z pair, sample name, and normalized ion intensity, were imported into SIMCA-P software (version 11.0, Umetrics, Umea, Sweden) for principle component analysis (PCA) and partial least squares discriminate analysis (PLS-DA). The variable importance in the projection (VIP) values were generated and represented the contribution to the intergroup discrimination of each metabolite ion. Metabolite ions with VIP values greater than 1.0 were selected for further analysis.

All data are presented as the means ± standard errors of the means (SEM). Statistical significance of the mean values was assessed using a one-way analysis of variance (ANOVA) or the Kruskal-Wallis H test as appropriate to assess differences among groups in SPSS 17.0 statistical software (SPSS Inc., Chicago, IL, United States) under the conditions of *p* < 0.05.

### Identification of Metabolites

The identification of potential urinary metabolites is important and challenging. Therefore, we identified the mass metabolites in a stepwise manner. First, we confirmed the ions based on the extracted ion chromatogram (EIC). Second, we input the accurate molecular mass of the ions into online databases, such as Metlin (http://metlin.scripps.edu/), Human Metabolome Database (http://www.hmdb.ca/), and the Mass Bank (http://www.massbank.jp/), to search for possible metabolites. Third, we compared the MS/MS spectra with the MS/MS information from databases to verify the structure of some putative metabolites.

### Network Analysis

The 15 main compounds of GS and their potential targets were acquired as described in our previous research (Hou et al., 2018). In the present study, the interacting proteins of differentially altered metabolites were further searched using the Search Tool for Interactions of Chemicals (STITCH, http://stitch.embl.de) database (Szklarczyk et al., 2016). Subsequently, the interacting proteins of differentially altered metabolites were pooled with the targets of components of GS to construct the component-target-metabolite network using Cytoscape software (http://cytoscape.org).

### Western Blot

Acquired renal tissues were homogenized in lysis buffer (KeyGEN, Nanjing, China) containing protease inhibitors and phosphatase inhibitors. The lysates were centrifuged at 12,000 rpm for 5 min at 4°C and the supernatant was collected. The protein concentrations of mixed lysates were determined using a BCA protein assay kit (Thermo Fisher Scientific). Equal amounts of total protein were separated on SDS-PAGE gels and transferred onto nitrocellulose membranes (GE Healthcare Life Sciences). After blocking, the membrane was incubated with rabbit polyclonal anti-ESR2 (1:1,000, Boster Biological Technology), anti-ESR1 (1:1,000, Santa Cruz Biotechnology), anti-AR (1:500, Cloud-Clone), anti-MAOB (1:1,000, Wuhan USCN), anti-PGR (1:1,000, Abcam), anti-GBA (1:1,000, Wuhan USCN), anti-THRB (1:1,000, Abcam), anti-ALB (1:1,000, Cell Signaling Technology), anti-CMA1 (1:1,000, Wuhan USCN) and anti-GAPDH (1:1,000, Wuhan USCN) antibodies at 4°C overnight. After washes with TBST, the membrane was incubated with a fluorescent dye-conjugated secondary anti-rabbit antibody (1:10,000, Licor) for 60 min at room temperature. The signals were visualized using the Odyssey Infrared Imaging System (Licor, NE, United States) and quantitatively analyzed by normalizing them to GAPDH levels using ImageJ software.

### Immunohistochemical Staining

For IHC staining, renal sections were incubated at 4°C overnight with primary antibodies against ESR2 (1:50; Cloud-clone, Wuhan, Hubei, China) and AR (1:50; Cloud-clone, Wuhan, Hubei, China), and then washed with PBS. The specimens were subsequently incubated with an HRP-conjugated goat anti-rabbit antibody (1:500, Proteintech, Chicago, IL, United States) at 37°C for 1 h. Next, the sections were stained with 3,3′-diaminobenzidine (DAB; Boster, Wuhan, Hubei, China) for 6 min.

## Results

### Quality Control of the GS Extract

**FIGURE 1:**
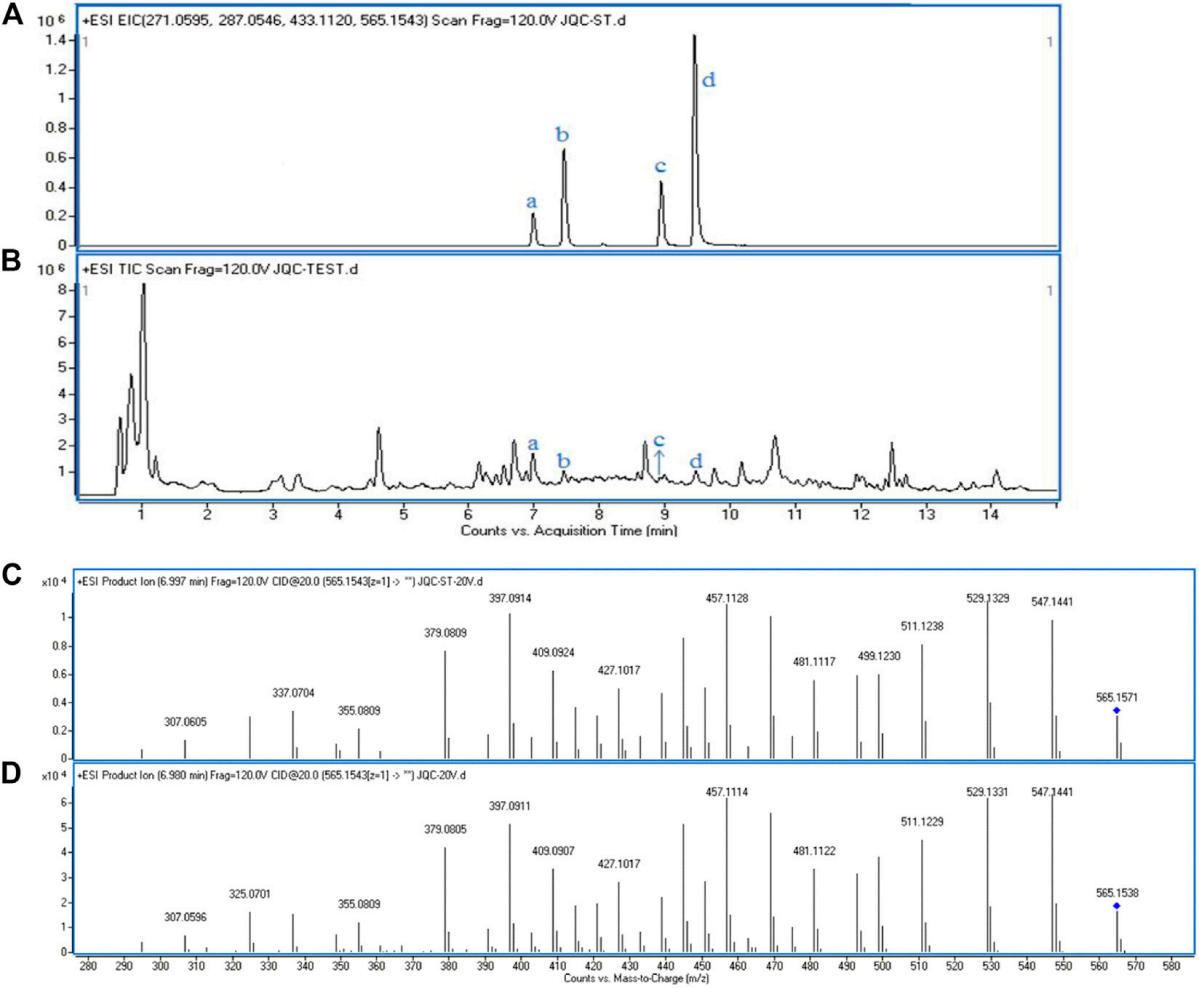
The EICs and the MS/MS spectrum of standard substances and GS extraction. **(A)** EICs of four standard substances in positive mode. **(B)** Total ion chromatogram of GS extraction in positive mode: a. Schaftoside, b. Isovitexin, c. Luteolin, d. Apigenin. Tandem spectrum of standard, Schaftoside **(C)** and GS extraction **(D)** in positive mode.

The GS was parallel-extracted six times, and an UHPLC-Q-TOF/MS approach was used to test the reproducibility of the extraction of GS. As shown in Figures 1A,B, the EIC of the four standard substances and the GS extraction visually showed a very similar profile of retention time, which demonstrated that all of these standard substances could be found in the GS extraction. Each RSD value of the peak area was below 3.2%, as shown in Table 1, which indicated that the UHPLC-Q-TOF system was stable and repeatable and that the extraction of GS was reproducible and reliable. Tandem mass spectrometry (MS/MS) was further performed and was shown to be matched between the standard substances and GS extraction (Figures 1C,D). More information about the MS/MS is provided in the Supplemental Materials section (Supplementary Figure S1).

**TABLE 1:** The peak area and RSD values of components.

Compound	Schaftoside	Isovitexin	Luteolin	Apigenin
Area^a^	4984479.83 ± 84153.52	2769191 ± 69812.83	381927 ± 12104.72	696599.5 ± 17078.66
RSD (%)	1.69	2.52	3.17	2.45

a The peak area of each standard substance.

### Cell Model and Treatment

LDH is a cytoplasmic enzyme that can be released outside when cells are damaged or dead. The number of injured and dead cells is proportional to the level of LDH released to the culture medium. As shown in Supplementary Figure S2A, the release of LDH in NaOx-treated cells was significantly higher than that in the control group. The treatment of GS and K3Cit (as a positive control) significantly inhibited the LDH release stimulated by oxalate. The secretion of TNF-α and IL-6 of the oxalate-stimulated HK-2 cells was significantly increased, compared with nontreated cells. However, the secretion of these cytokines was significantly inhibited by the treatment of GS and K3Cit (Supplementary Figures S2B,C).

### Biochemical Analysis

**FIGURE 2:**
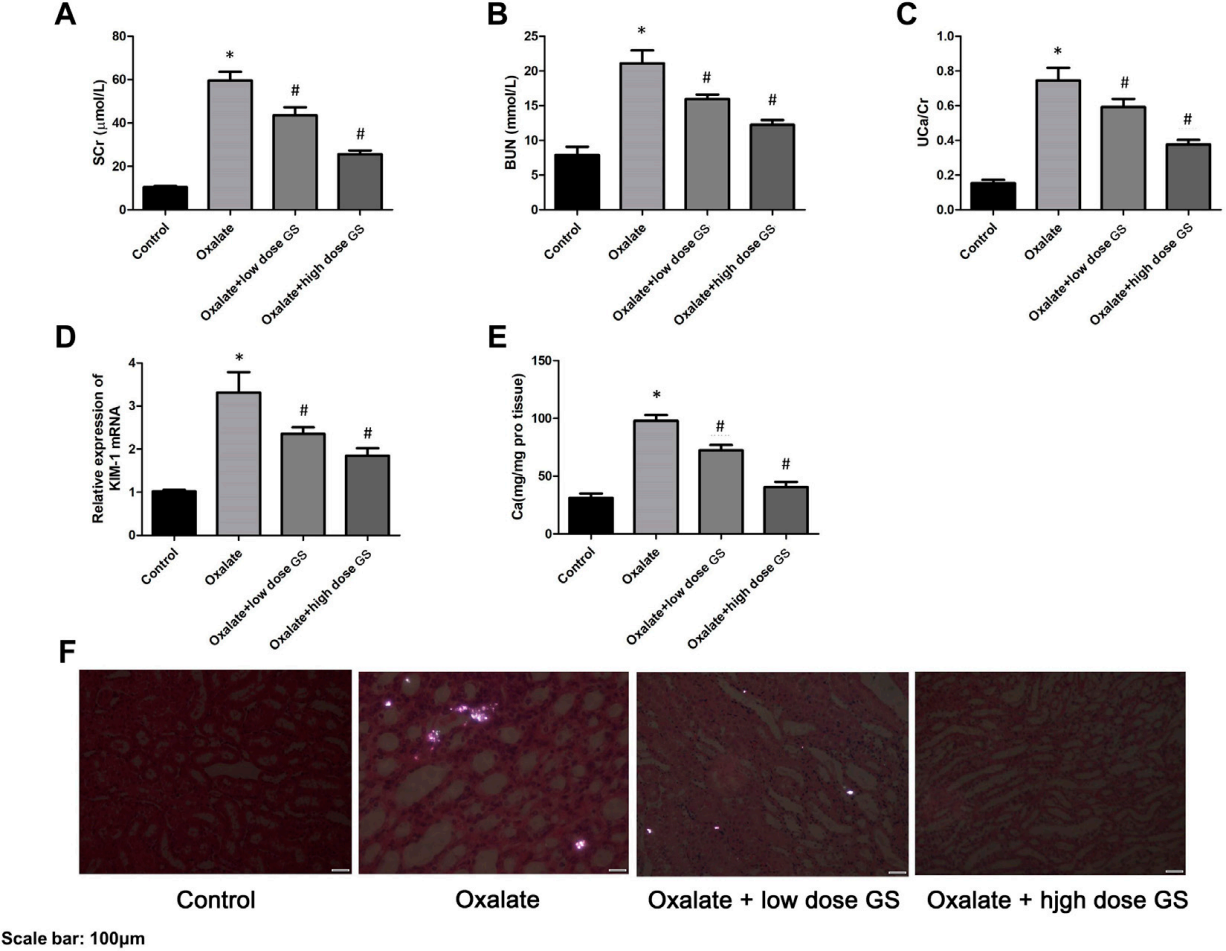
GS protected renal function and alleviated crystal deposition. The levels of SCr **(A)** and BUN **(B)** in the serum, UCa/Cr **(C)** in the urine, renal KIM-1 mRNA **(D)** and calcium contents **(E)** in the renal tissues in the control group, oxalate group, oxalate + low-dose GS group, and oxalate + high-dose GS group. **(F)** Detection of renal CaOx crystal deposition in the four groups with a polarizing microscope. The results are presented as means ± SEM. Statistical comparisons were performed using a Newman-Keuls test (*p < 0.05 compared with the control group and ^#^p < 0.05 compared with the oxalate group).

The levels of SCr, BUN, UCa/Cr, renal KIM-1 mRNA and the calcium contents in renal tissues in the oxalate group were markedly increased compared to the control group, but were significantly reduced by the GS treatment, especially at a high dose (Figures 2A–E).

### Detection of Crystal Deposition

Using a polarizing microscope, CaOx crystal deposition was clearly present in the renal corticomedullary junction in the oxalate group, while the crystal deposition was obviously alleviated by the GS treatment, especially at a high dose (Figure 2F).

### Metabolic Profile of Urine

**FIGURE 3:**
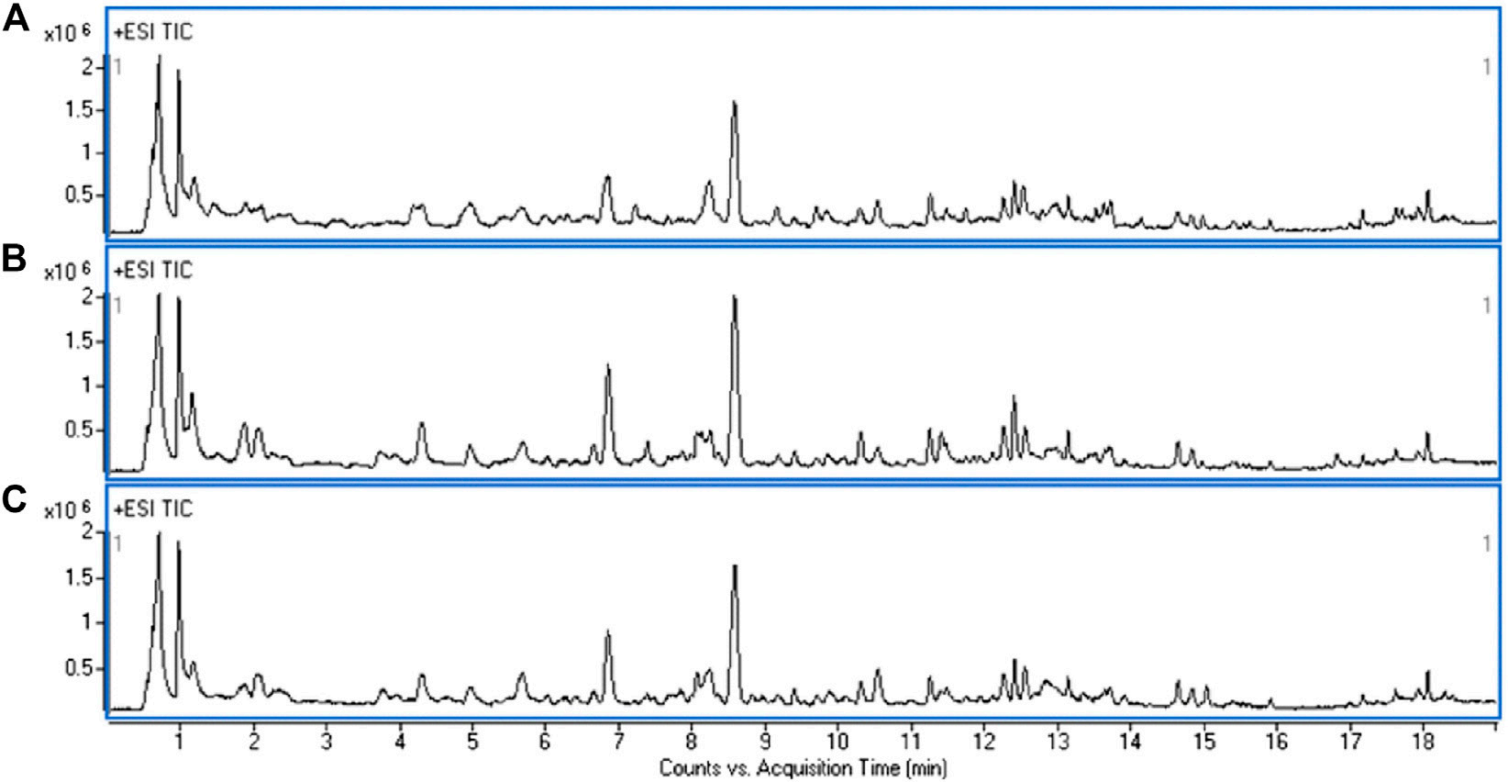
Representative TIC obtained for the ESI-positive ion in the control group **(A)**, oxalate group **(B)** and oxalate + high-dose GS group **(C)**.

**FIGURE 4:**
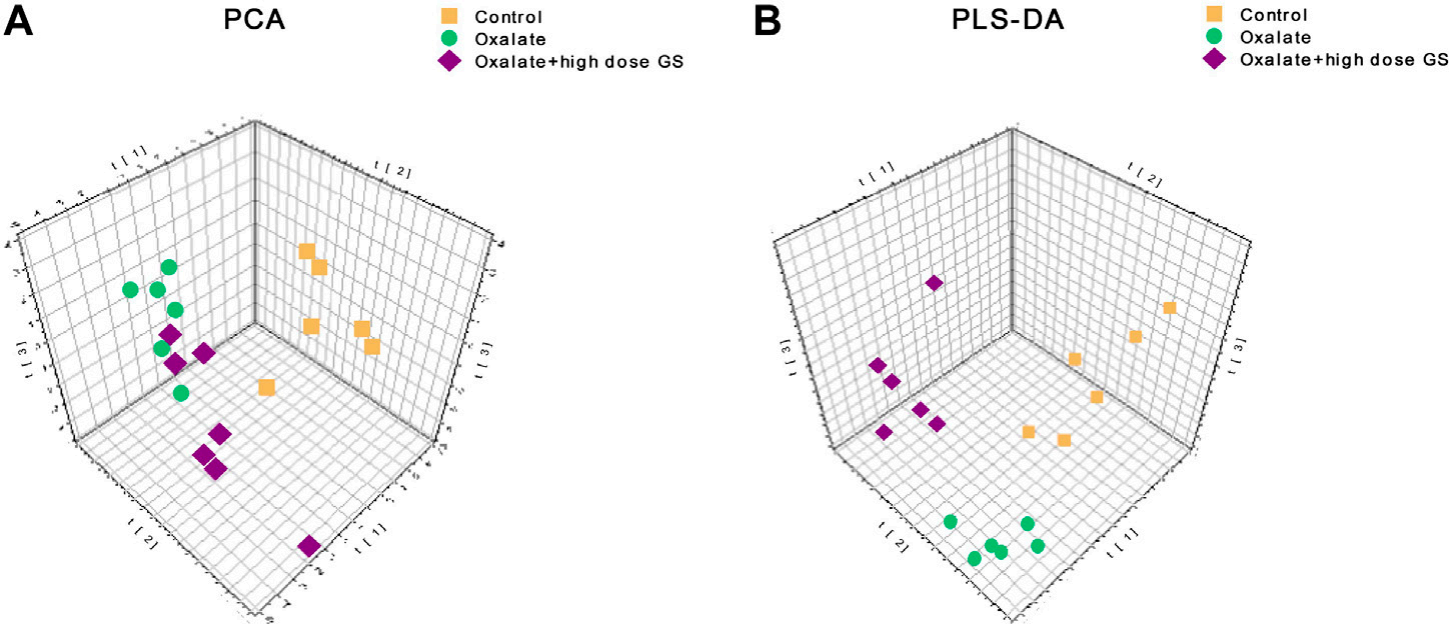
PCA and PLS-DA score plots in ESI positive ion mode. **(A)** PCA score plots based on the urine metabolic profiles of the control group, oxalate group and oxalate + high-dose GS group. **(B)** PLS-DA score plots of urine samples obtained from the control group, oxalate group and oxalate + high-dose GS group.

A UHPLC-Q-TOF/MS analysis was performed in both positive and negative modes to explore the metabolic changes in the pathological process of oxalate-induced kidney injury and the mechanism of the protective effects of high-dose GS. As shown in the total ion chromatogram (TIC) and the corresponding spectra collected in positive mode of the control group, oxalate group and oxalate + high-dose GS group were similar (Figure 3 and Supplementary Figure S3). After preprocessing the data, PCA was performed to extract systematic variations and to visualize the general relationships between the control group, oxalate group and oxalate + high-dose GS group (Figure 4A and Supplementary Figure S4A). Furthermore, PLS-DA was applied to screen the potential metabolites, and the three-dimensional score plot showed that the oxalate group was obviously separated from the control group (Figure 4B and Supplementary Figure S4B). However, the oxalate + high-dose GS group was close to the control group, revealing that GS rectified the metabolic deviations and protected against crystal-induced kidney injury.

**FIGURE 5:**
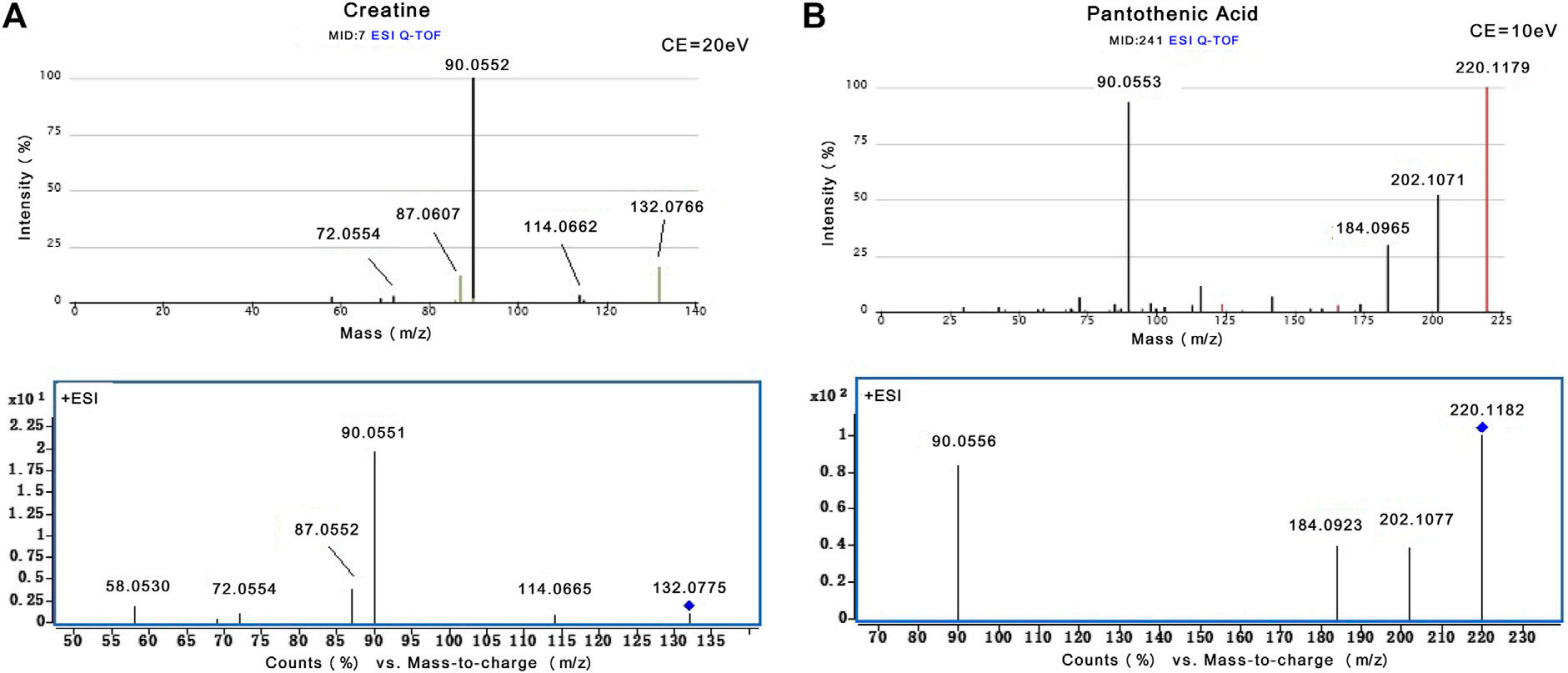
The identification of creatine and pantothenic acid. **(A)** MS/MS spectra of creatine from Metlin database and actual collection (m/z 132.08). **(B)** MS/MS spectra of pantothenic acid from Metlin database and actual collection (m/z 220.12).

**FIGURE 6:**
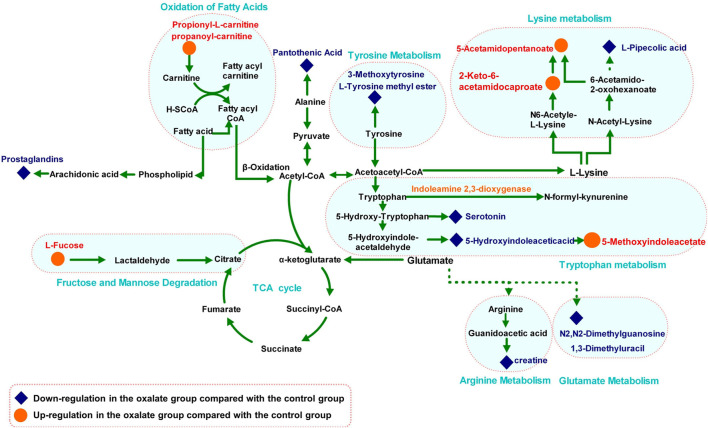
Metabolic pathway network related to oxalate crystal-induced kidney injury. The levels of metabolites in the oxalate group compared with those in the control group are labeled in red (up-regulated) and blue (down-regulated).

The MS-based identification of the potential metabolites was conducted using online databases. Fifteen metabolites that were differentially present between the control group and oxalate group were finally identified as differentially altered metabolites associated with crystal-induced renal injury (Table 2). Among these metabolites, creatine and pantothenic acid were verified based on the MS/MS spectra (Figure 5). These fifteen endogenous metabolites were mainly involved in amino acid metabolism and fatty acid metabolism. A metabolic network covering all of the differentially altered metabolites was constructed to comprehensively understand the metabolic disturbances that accompanied the occurrence of crystalline nephropathy (Figure 6). In addition, these differentially altered metabolites were further analyzed using network pharmacology.

**TABLE 2:** The differentially altered metabolites related to oxalate-induced nephrotoxicity and their metabolic pathways.

Class	No	Rt/min	Quasi-molecular ion (m/z)^a^		Ions	Error (ppm)	Formula	Metabolite	Assignment based on	VIP	Fold change^b^	
			Observed	Theoretical							Oxalate/Control	High dose GS/Oxalate
Amino acids	1	0.68	132.0775	132.0768	[M + H]+	5	C_4_H_9_N_3_O_2_	Creatine	AM^c^, TM^d^	1.76	^e^0.72	^e^1.94
	2	0.85	130.086	130.0863	[M + H]+	1	C_6_H_11_NO_2_	L-Pipecolic acid	AM	1.31	^e^0.85	1.37
	3	8.03	194.0809	194.0823	[M-H]-	7	C_10_H_13_NO_3_	L-Tyrosine methyl ester	AM	2.16	^e^0.36	^e^976.00
	4	4.81	212.092	212.0917	[M + H]+	1	C_10_H_13_NO_4_	3-Methoxytyrosine	AM	1.45	^e^0.42	1.03
Organic acids and derivatives	5	3.83	188.0918	188.0917	[M + H]+	0	C_8_H_13_NO_4_	2-Keto-6-acetamidocaproate	AM	1.05	^e^1.68	1.05
Aromatic heteromonocyclic compounds	6	0.9	141.0658	141.0659	[M + H]+	0	C_6_H_8_N_2_O_2_	1,3-Dimethyluracil	AM	1.66	^e^0.40	1.78
Aromatic heteropolycyclic compounds	7	7.49	214.0477	214.0475	[M + Na]+	1	C_10_H_9_NO_3_	5-Hydroxyindoleacetic acid	AM	1.77	^e^0.69	5.08
	8	10.53	228.0634	228.0631	[M + Na]+	1	C_11_H_11_NO_3_	5-Methoxyindoleacetate	AM	1.11	^e^2.44	2.27
	9	1.42	177.1018	177.1022	[M + H]+	2	C_10_H_12_N_2_O	Serotonin	AM	1.58	^e^0.36	1.3
Lipids	10	1.01	182.0787	182.0788	[M + Na]+	0	C_7_H_13_NO_3_	5-Acetamidopentanoate	AM	1.79	^e^3.91	0.29
	11	1.21	153.0654	153.0659	[M + H]+	0	C_7_H_8_N_2_O_2_	Prostaglandins	AM	1.13	^e^0.81	^e^1.06
	12	6.37	218.1389	218.1387	[M + H]+	0	C_10_H_19_NO_4_	Propanoyl-carnitine	AM, +/−^f^	1.41	^e^1.82	0.81
Nucleosides	13	2.85	312.1308	312.1302	[M + H]+	0	C_12_H_17_N_5_O_5_	N_2_,N_2_-dimethylguanosine	AM	1.26	^e^0.56	1.41
Carbohydrates and carbohydrate conjugates	14	0.69	163.0601	163.0612	[M-H]-	6	C_6_H_12_O_5_	L-fucose	AM	1.93	^e^2.84	^e^0.552
Aliphatic acyclic compounds	15	2.45	220.1182	220.1179	[M + H]+	0	C_9_H_17_NO_5_	Pantothenic acid	AM, TM	1.58	^e^0.67	1.37

a Quasi-molecular ions (m/z) in this table include three species, namely, [M + H]+ or [M + Na]+ (ES + mode) and [M−H] − (ES− mode).

b Statistical comparisons were performed using a Tukey post hoc test.

c Accurate mass.

d Tandem mass spectrometry.

e *p* < 0.05.

f ESI^+^ and ESI^−^ mode cross-assist analysis.

Among the 15 metabolites associated with renal injury, the trends of 13 metabolites were reversed by the GS intervention. The level of L-tyrosine methyl ester decreased after oxalate stimulation and displayed a significant tendency to be reversed by GS treatment. In addition, levels of L-fucose increased considerably in response to oxalate stimulation and exhibited a significant tendency to be corrected to a normal level after GS treatment.

### Network Pharmacology Analysis

**FIGURE 7:**
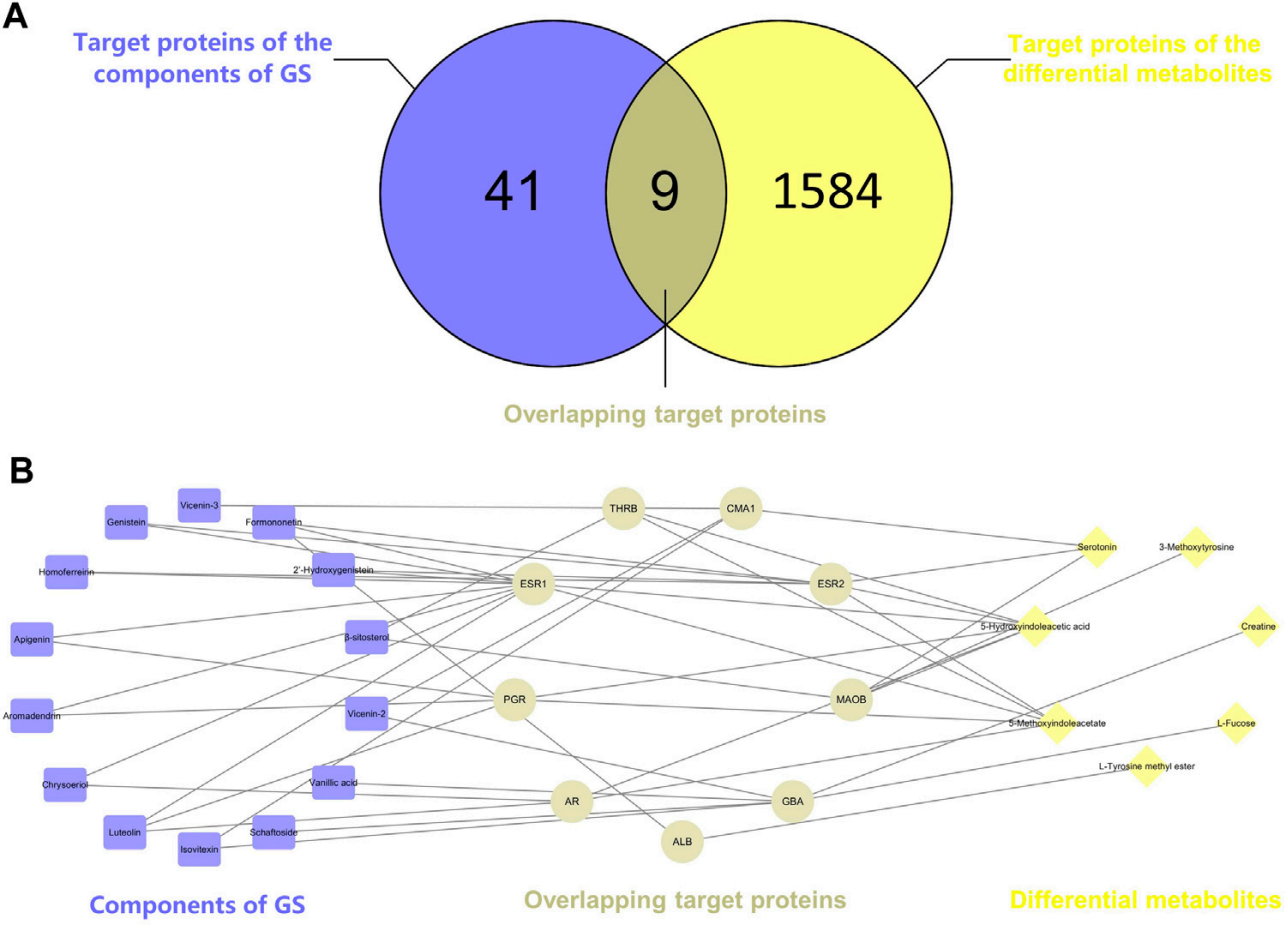
**(A)** Venn diagram. The intersection of two circles represents the overlapping target proteins of the components of GS and the differentially altered metabolites. **(B)** The component-target-metabolite network in the treatment of oxalate-induced kidney injury by GS. The purple nodes (14) represent the components of GS, the yellow nodes **(C)** represent the differentially altered metabolites, and the gray nodes **(D)** represent the overlapping proteins.

Potential targets of the components of GS were previously acquired using PharmMapper Server (Hou et al., 2018). Proteins that interacted with differentially altered metabolites were obtained from the STITCH database (Supplementary Table S1). Nine overlapping related proteins interacting with GS and metabolites were identified by constructing a Venn diagram (Figure 7A). A component-target-metabolite network was constructed with Cytoscape software to visually reveal the interactions among the components of GS, target proteins and differentially altered metabolites (Figure 7B and Supplementary Table S2). In the network, nine overlapping target proteins, including ESR2, ESR1, AR, MAOB, PGR, GBA, THRB, ALB and CMA1, were identified for further analysis. This network indicated that the components of GS might regulate multiple metabolic pathways through multiple targets to exert various therapeutic effects.

### Validation of Potential Targets

The nine overlapping proteins were further verified by western blotting. As illustrated in Supplementary Figure S5, a significantly decreased level of ESR2 and increased level of AR were observed in the oxalate group. These changes were reversed by the GS treatment. However, the expression of the other seven overlapping proteins was not significantly different.

**FIGURE 8:**
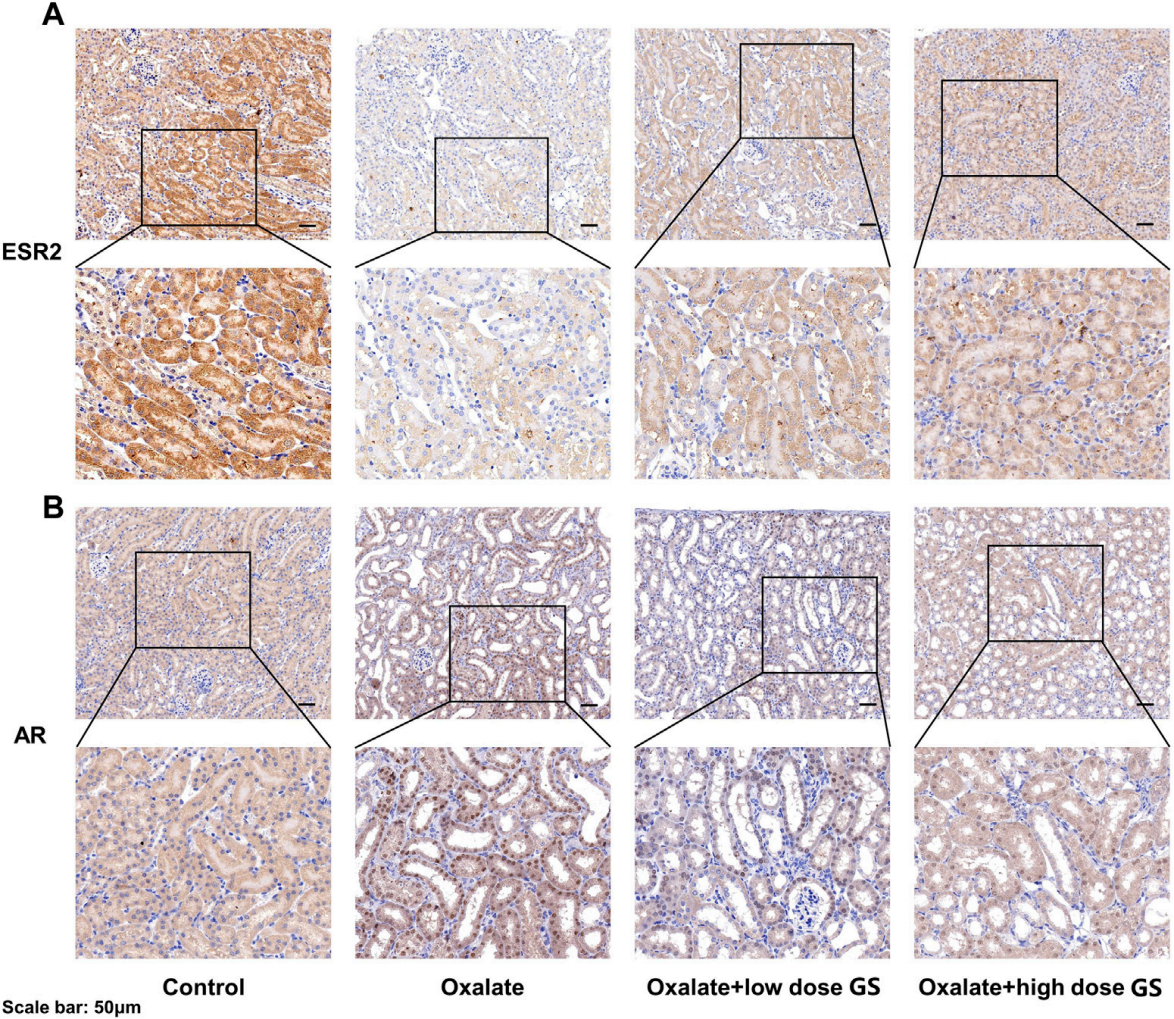
The expression of ESR2 **(A)** and AR **(B)** in the renal tissues from the control group, oxalate group, oxalate + low-dose GS group, and oxalate + high-dose GS group detected using IHC staining.

The expression of ESR2 and AR was further investigated using IHC staining, and the expression of ESR2 in the tubular cells was significantly decreased in the oxalate group compared with the control group, but was increased by the GS treatment, especially at a high dose (Figure 8A). In addition, AR expression in the nuclei of tubular cells was obviously increased in the oxalate group compared to the control group, and this change was reversed by GS, especially at a high dose (Figure 8B).

## Discussion

### Amino Acid Metabolism

Amino acid metabolism participates in the processes of protein synthesis and degeneration, energy supply, gluconeogenesis, ketogenesis and biosynthesis of some signaling molecules. In the model group, the levels of several amino acids and their endogenous relative metabolites were noticeably changed. In our study, the involved pathways, including tyrosine metabolism, lysine metabolism, tryptophan metabolism, arginine metabolism and glutamate metabolism, were substantially altered.

As an essential amino acid in mammals, tryptophan was shown to play a notable role in the pathological processes of many kidney diseases, such as chronic kidney disease and nephrotoxicity, because it can be further transformed into many biological molecules and has an important function in homeostasis (Bossola and Tazza, 2015). In the model group, levels of L-tyrosine methyl ester, serotonin and 5-hydroxyindoleacetic acid, the endogenous biological derivative of tryptophan, were reduced (Fernandez-Canon et al., 2002). Based on our present work, we propose that the metabolism of these amino acids is inseparably connected with oxalate-induced renal injury. Levels of all of these amino acids were restored to the normal level after the intervention with GS, indicating that GS has therapeutic efficacy in maintaining normal tryptophan metabolism.

Tyrosine is a semi-essential amino acid in our body, along with tryptophan, and it is also an aromatic amino acid that has been documented to be associated with renal diseases (Zhan et al., 2002). In the model group, the levels of 3-methoxytyrosine and L-tyrosine methyl ester were significantly decreased. In contrast, GS treatment significantly increased the levels of those metabolites, suggesting that the disturbed metabolic state in the urine from mice with oxalate-induced crystal-related kidney injury was in the process of returning to normal after the GS intervention.

Both 1, 3-dimethyluracil and N2, N2-dimethylguanosine are metabolites of glutamic acid and were reduced in the model group. Meanwhile, they were shifted toward the baseline level in the control group after the GS treatment. Glutamic acid was recognized many years ago as a basic amino acid participating in protein biosynthesis that is converted into α-ketoglutarate through multiple pathways, and it is an essential metabolic intermediate in the tricarboxylic acid cycle (TCA) (Curthoys, 2001). Therefore, the changes in the levels of 1, 3-dimethyluracil and N2, N2-dimethylguanosine may indicate that energy metabolism was affected during the pathological process.

L-arginine plays important roles in the body, as it participates in the disposal of ammonia and is converted to glucose and glycogen under conditions of an inadequate energy supply. Furthermore, arginine is also the precursor of creatine in the urea cycle (Karamat et al., 2015). Levels of serotonin, creatinine and pantothenic acid, the metabolic derivatives of arginine, were decreased in the crystal kidney injury group. Arginine is transformed into acetyl-coenzyme A through pyruvate, which acts as the starting point for a series of physiological reactions known as the TCA (Schneider et al., 2011). The abnormal levels of these metabolites were rectified by GS, which further normalized amino acid metabolism. Therefore, we have many reasons to speculate that the therapeutic efficacy of the targeted GS intervention against crystal-induced kidney injury is mainly mediated by normalizing the disordered TCA through changes in the arginine and glutamate metabolic pathways.

### Fatty Acid Metabolism

Fatty acid metabolism has a direct relationship with the energy supply in organisms through fat mobilization and β-oxidation. In this process, L-carnitine is a key factor because it helps transport long-chain fatty acids into the mitochondrial matrix for the utilization of fatty acids through β-oxidation (Knottnerus et al., 2018). Therefore, an increased intracellular level of carnitine provides an additional energy supply. In the model group, levels of propionyl-L-carnitine, a product of the enzymatic esterification of carnitine, increased significantly, which indicated the presence of a pathological process induced by the glyoxylate intervention. The subsequent CaOx deposition pushed the organism into a high metabolic state to satisfy the energy requirement by increasing the β-oxidation process in the mitochondria. However, large quantities of reactive oxidants were produced during the process of β-oxidation, leading to severe oxidative injury to the renal epithelial cells. Following treatment with GS, the increased propionyl-L-carnitine level was significantly reduced, suggesting that the disordered fatty acid metabolism induced by glyoxylate was in the process of returning to a normal state.

### Component-Target-Metabolite Network Analysis

According to the network construction, we identified nine overlapping proteins might play important roles in the therapeutic effects of GS on CaOx crystal-induced renal injury. Interestingly, ESR1, ESR2 and AR are the predicted targets of the components of GS among these overlapping targets. Since nephrolithiasis is more common in men than in women, sex hormones and their receptors may play important roles in kidney stone formation and CaOx crystal-induced renal injury. Estrogen has been indicated to protect against kidney stone formation by lowering urinary CaOx saturation through a decrease in the urinary excretion of calcium and oxalate (Heller et al., 2002). In addition, ESR2 was reported to suppress renal CaOx crystal deposition by reducing hepatic oxalate biosynthesis and to alleviate crystal-induced injury by inhibiting reactive oxygen species production (Zhu et al., 2019b). However, androgen and AR signaling may promote kidney stone formation and aggravate renal injury since the plasma testosterone level and AR expression in the kidneys are up-regulated in men with nephrolithiasis (Li et al., 2010). Similarly, a previous study suggested that AR increased CaOx crystal deposition by increasing oxalate biosynthesis and oxidative stress (Liang et al., 2014). Moreover, the loss of AR suppressed renal CaOx crystal deposition by promoting M2 macrophage recruitment/polarization (Zhu et al., 2019a). In our study, ESR2 expression in renal tissues was down-regulated while the AR expression in the nuclei of tubular cells was increased in the oxalate group compared with the control group, changes that were reversed by GS. Therefore, we speculated that GS might exert its protective effect on CaOx crystal-induced renal injury by activating ESR2 and decreasing the nuclear translocation of AR.

## Conclusion

**FIGURE 9:**
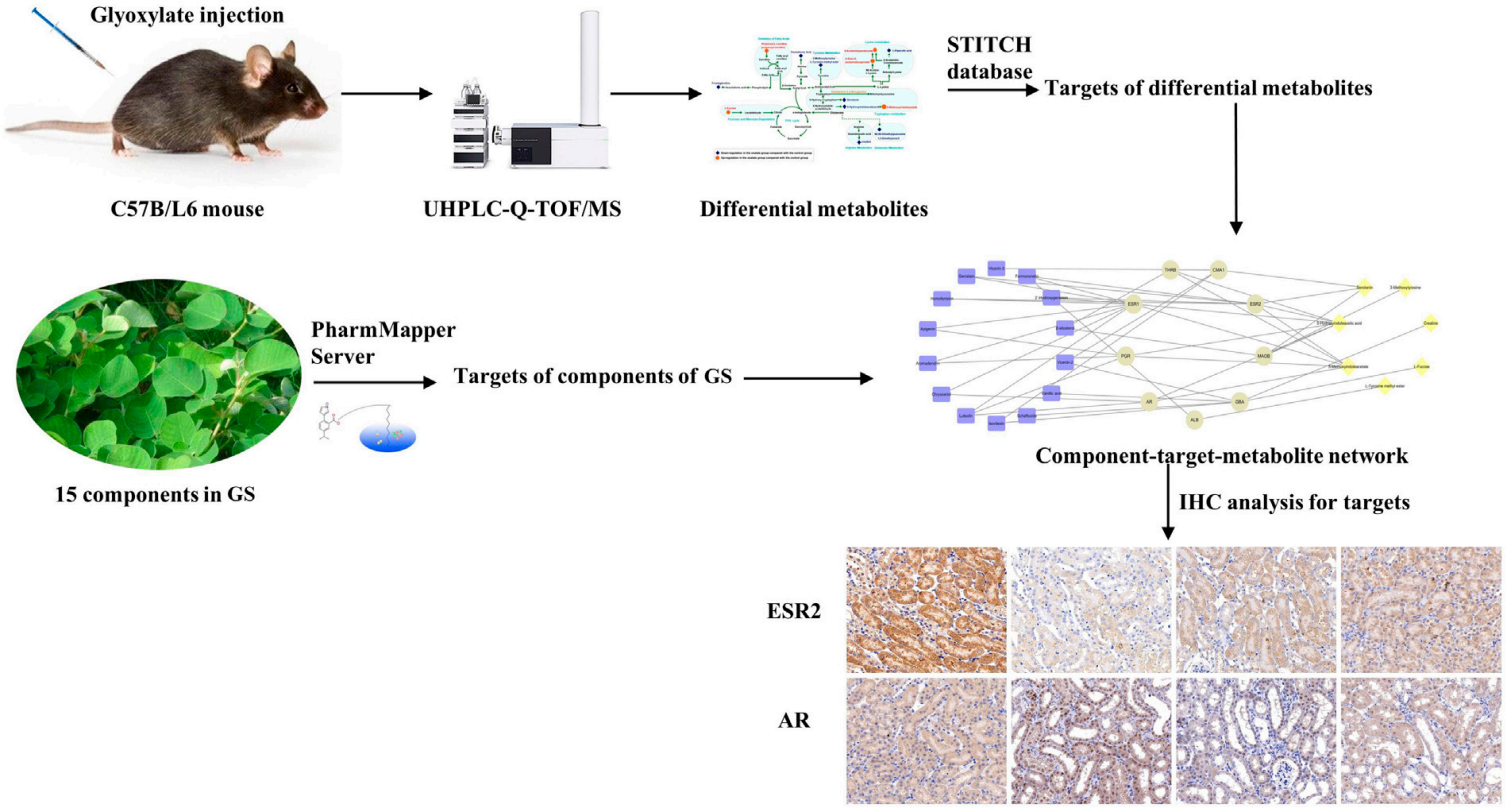
Roadmap of this study.

In the present study, GS was verified to alleviate renal injury induced by CaOx. The roadmap of this study is shown in Figure 9. We employed an integrative pharmacology-based strategy combining network pharmacology and metabolomics to decipher the underlying mechanism. Fifteen metabolites involved in amino acid and fatty acid metabolism were identified as potential biomarkers of crystal-induced renal damage and the changes in the levels of 13 metabolites were reversed by the GS intervention. By integrating network pharmacology, a component-target-metabolite network was constructed. Among the nine overlapping target proteins, two crucial targets (ESR2 and AR) were differentially expressed in the kidney of oxalate group, but these trends were reversed by the GS treatment. In conclusion, GS exerts its therapeutic effect by regulating multiple metabolic pathways and the expression of ESR and AR in mice with oxalate-induced renal injury. Our findings might help us consider novel potential targets when treating nephrolithiasis and obtain a better understanding of the mechanism underlying the curative effect of GS.

## Data Availability

The raw data supporting the conclusion of this article will be made available by the authors, without undue reservation.

## Abbreviations

CaOx, calcium oxalate; GS, *Grona styracifolia* (Osbeck) Merr; H. Ohashi and K. Ohashi; UHPLC-Q-TOF/MS, ultra-high-performance liquid chromatography coupled with quadrupole-time-of-fight mass spectrometry; MS/MS, tandem mass spectrometry; RSD, relative standard derivation; ESI, electrospray ionization source; PCA, principle component analysis; PLS-DA, partial least squares discriminate analysis; VIP, variable importance in the projection; EIC, extracted ion chromatogram; TIC, total ion chromatogram; ESR2, estrogen receptor 2; AR, androgen receptor.
