# Supplementary material for: Metabolic and Network Pharmacological Analyses of the Therapeutic Effect of Grona styracifolia on Calcium Oxalate-Induced Renal Injury
Source: Front Pharmacol. 2021 Jun 25;12:652989. doi: 10.3389/fphar.2021.652989 (PMC8267468; doi:10.3389/fphar.2021.652989)
Supplement: Supplementary file 1 [file DataSheet1.docx]

**Method S1. The quality control of the GS extract.**

The extraction of GS was repeated to acquire six samples. In this experiment, UHPLC-Q-TOF/MS was used to analyse the quality of the GS extract. The detailed method was at below:

UHPLC-Q-TOF/MS analysis was performed on Agilent 1290 Infinity LC system equipped with a Quadrupole Time-of-Flight mass spectrometer (Sciex TripleTOF). Liquid chromatography separation was performed at 30°C on a Agilent SB- C18 (2.1×150mm, 1.8μm) column. The mobile phase consisted of 0.1 % formic acid (A) and methanol (B). The optimized UPLC elution conditions were: 0-10min, 25%B; 10-22min, 30%B; 22-26min, 50%B; 26-30min, 60%B; 30-36min, 95%B; 36-40min, 25%B, and the post time was set to 6 min for equilibrating the system. The flow rate was set to 0.3 ml/min and the injection volume was 5μL. Each sample was injected once, and the corresponding relative standard deviation (RSD) value was calculated. The RSD is defined as the ratio of the standard deviation to the mean and often expressed as a percentage. The masss pectra were acquired in a range of 100 to 1500. The negative and positive electrospray ionization modes were applied for the mass spectrometer, and the collision energy was set from -40eV to 40 eV. Major constituents of GS, including schaftoside, luteolin, and apigenin, were further analyzed and verified by MS/MS.

**Supplementary Table S1. Target proteins of the differential metabolites.**

| **Differential metabolite** | **Target proteins** | | | | |
| --- | --- | --- | --- | --- | --- |
| Pantothenic acid | Vnn1 | Pank1 | Pank2 | Pank3 | Pank4 |
|  | Ppcdc | Coasy | Dcakd | Serpine2 | Hps3 |
|  | Slc5a6 | Pde6b | Slc23a2 | Vnn3 | Slc23a1 |
|  | Ppcs | Rps17 | Gm468 | Ppara | Slc5a12 |
|  | Slc5a8 | Slc5a5 | Slc23a3 | Ercc3 | Rps6ka2 |
|  | Ccbl2 | Ccbl1 | Vps13d | Pqbp1 | Krt71 |
|  | Ndufs3 | Dhrs11 | Atxn7 | Atp5a1 | Mrpl2 |
|  | Sry | Cd8a | Vps13a | Smc2 | Tufm |
|  | Gm9755 | Chchd7 | Upb1 | Fabp1 | Pdxk |
|  | Pm20d1 | Snf8 | Gad1 | Aldh18a1 | Atxn7l1 |
|  | Fabp7 | Rcl1 | Clpp | Il2ra | Rusc1 |
|  | Fdps | Vps13c | Acy1 | Acot13 | Mkrn3 |
|  | Zfp57 | Rps6ka1 | Rps6ka6 | Thsd1 | Rps6ka3 |
|  | Nnt | Slc25a31 | Dcaf17 | Slc25a4 | Slc25a5 |
|  | Cd4 | Mut | Mtbp | Krt74 | Vars |
|  | Vars2 | Akr1c18 | Lgals4 | Vbp1 | Ist1 |
|  | Gm4076 | mt-Nd5 | Eepd1 | Rps6kb1 | Pold2 |
|  | mt-Co2 | Gpt2 | Rps6kb2 | Gpt | Pemt |
|  | ENSMUSG00000055312 | Synm | Tmem131 | Fcf1 | Bckdk |
|  | Pdk2 | Pdk3 | Pdk4 | Pdk1 | Polr2c |
|  | Polr1c | Stt3a | Stt3b | Mkrn1 | Eif2ak4 |
|  | Aadat | Amt | Tmprss11d | Gad2 | Ndufs2 |
|  | Gstm7 | Uprt | Uckl1 | Aass | Ctnnb1 |
|  | Jup | Decr1 | Adsl | Stau1 | Hspbp1 |
|  | Tmem67 | Steap4 | Steap3 | Mmab | Steap2 |
|  | Siah2 | Engase | Pars2 | Dhodh | Eprs |
|  | Lepr | AI597479 | Ctps2 | Ctps | Glud1 |
|  | Enpp1 | Vps13b | Ndufab1 | Fgf21 | Coq3 |
|  | Enpp3 | Msrb1 | Lig1 | Msrb3 | Fdft1 |
|  | Msrb2 | Nudt7 | Tubb4b | Dpp3 | Ap1m2 |
|  | Usp32 | Orc1 | Cdc6 | Mudeng | Ap4m1 |
|  | Ap2m1 | Ap1m1 | Abo | BC017158 | Pmp2 |
| L-Fucose | Fuk | Fuca1 | Fuca2 | Fxyd1 | Serpind1 |
|  | Dhx9 | Mtif2 | B3galt6 | Alg2 | Dhx36 |
|  | Arsa | Arsg | Galns | Pars2 | Eprs |
|  | Galk2 | Galk1 | Prkcsh | Alg11 | Sod3 |
|  | Sod1 | Miox | Fam53b | Gnptg | Pigm |
|  | Mef2c | Mef2a | Mef2b | ENSMUSG00000002345 | Mef2d |
|  | Serpinc1 | Gbe1 | Sar1b | Sar1a | Dera |
|  | Ids | Arsj | Arsb | Arsi | Eif5b |
|  | Sgsh | Dhx57 | Dhx30 | Dhx29 | Fut8 |
|  | Tpi1 | Add1 | Add2 | Add3 | Apip |
|  | Aldh3a2 | Aldh3a1 | Plg | Pofut2 | D2hgdh |
|  | Ldhd | Gulo | A4gnt | Agps | Dhcr24 |
|  | Fuom | Cpa5 | Fut4 | Gtdc2 | Gmds |
|  | Tdrd9 | Ythdc2 | Arhgap44 | Gm4076 | mt-Nd5 |
|  | Tmem213 | Atp6v1d | Gcnt4 | Mpi | Lipk |
|  | Lipa | Mmab | Lipm | Lipf | Colec10 |
|  | Galnt11 | Masp1 | Hdhd1a | Srf | Pigt |
|  | Pigp | Prpf40a | Tmem52b | Mri1 | Mgat4c |
|  | St3gal6 | St6galnac4 | St3gal1 | St6gal2 | St6galnac2 |
|  | St8sia5 | St6galnac6 | St3gal5 | St6galnac3 | St8sia4 |
|  | St6galnac5 | St3gal4 | St3gal2 | St8sia1 | Tmprss11d |
|  | St3gal3 | St8sia2 | St8sia3 | St6gal1 | St6galnac1 |
|  | St8sia6 | Arhgap17 | Sh3bp1 | Athl1 | Adam7 |
|  | Pofut1 | Sulf1 | Sulf2 | Gns | Eogt |
|  | Fpgt | Pigs | Igf2r | Cyb5r4 | Glb1l |
|  | Cyb5rl | Glb1 | Cyb5r2 | Glb1l2 | Glb1l3 |
|  | Cyb5r1 | Oxnad1 | Cyb5r3 | Prpf40b | Lgals7 |
|  | Atp5o | Arsk | Polg | B3gnt6 | B3gnt3 |
|  | Prep | Sftpa1 | Actl9 | Gm498 | Actl10 |
|  | Actb | Actl7b | Actl7a | Actc1 | Actl11 |
|  | Gba | Actg2 | Actg1 | Clec12a | Actrt1 |
|  | Actbl2 | Uckl1 | Actrt2 | Arpm1 | Acta2 |
|  | Actr1b | Actr1a | Acta1 | Actr10 |  |
| Serotonin | Htr1a | Htr7 | Htr2a | Htr2c | Htr1b |
|  | Htr2b | Htr4 | Htr1d | Htr1f | Htr5a |
|  | Htr6 | Drd2 | Htr3a | Slc6a4 | Htr5b |
|  | Oprm1 | Oprd1 | Tbxa2r | Drd3 | Cnr1 |
|  | Ntsr2 | Ntsr1 | Drd4 | Drd1a | Opn4 |
|  | Mchr1 | Drd5 | Mc1r | Npbwr1 | Adra2c |
|  | Hrh2 | Adra1a | Mc5r | ENSMUSG00000029917 | Ccr9 |
|  | Ptgir | Ccr5 | Adora2a | Adra1d | Npy2r |
|  | Ptgdr | Qrfpr | Agtr2 | Adrb3 | Gpr65 |
|  | Adra2b | Oprl1 | Ccr1l1 | Agtr1a | Adra1b |
|  | Cnr2 | Cxcr7 | Sstr2 | Agtr1b | Galr1 |
|  | Npy5r | Rxfp3 | Ccr4 | Adrb2 | Ghsr |
|  | Cxcr6 | Prokr1 | Rxfp4 | Mc2r | Hcrtr2 |
|  | Sstr3 | Prokr2 | Ppyr1 | Npsr1 | Mc4r |
|  | Galr2 | Aplnr | Cxcr4 | Lpar1 | Ltb4r1 |
|  | Oxtr | Sstr5 | Hrh3 | Ccr2 | Taar1 |
|  | Ptger4 | Ltb4r2 | Mc3r | Uts2r | Npy1r |
|  | Bdkrb1 | Nmur2 | Sstr4 | Adora1 | Ptger3 |
|  | Hrh4 | Adrb1 | Kiss1r | Npffr2 | Ccr3 |
|  | Ptger2 | Lpar3 | Sstr1 | Trhr | Adra2a |
|  | Lpar2 | Ednra | Avpr2 | Grpr | Cckbr |
|  | Gnrhr | Cckar | Hcrtr1 | Ptgfr | Avpr1b |
|  | Nmur1 | Oprk1 | Ccr1 | Ednrb | Avpr1a |
|  | Npffr1 | Nmbr | Ptger1 | Adora2b | Tph1 |
|  | Maoa | Crhr1 | Pomc | Ghrh | Npy |
|  | Nps | Rgs20 | Gng2 | Adcy2 | Oxt |
|  | Calca | Rgs7 | Crhr2 | Slc18a2 | Sst |
|  | Rgs6 | Cck | Vip | Adcy7 | Ddc |
|  | Adcy4 | Maob | Kng2 | Kng1 | Gast |
|  | Hcrt | Tph2 | Gnrh1 | Pdyn | Tac1 |
|  | Rgs4 | Crh | Nts | Hrh1 | Gcg |
|  | Slc18a1 | Chrm2 | Adcy5 | Adcy6 | Grm2 |
|  | Adcy8 | Adcy3 | Adcy1 | ENSMUSG00000095585 | Gm2023 |
|  | Asmt | Gm1987 | Grm7 | Rgs1 | Grm4 |
|  | Gpr81 | Grm5 | Sct | Gm2442 | Aanat |
|  | Saa1 | Rgs2 | Ido2 | S1pr5 | ENSMUSG00000096506 |
|  | Grm8 | Kalrn | Glp1r | Ccl20 | Cxcl9 |
|  | Plcb1 | Npb | Plcb2 | Gip | Ccr7 |
|  | Chrm5 | Ccl28 | Ccr6 | Fpr3 | Fpr-rs4 |
|  | Fpr-rs7 | Fpr-rs6 | Npw | Tas2r121 | Tas2r115 |
|  | Tas2r140 | Gipr | Ffar3 | Trio | Lpar5 |
|  | Nms | S1pr3 | Gpr55 | Tas2r131 | Tas2r110 |
|  | Gpr30 | Tas2r114 | Gnas | Pth | Tas2r113 |
|  | Xcr1 | Rxfp1 | Gpbar1 | Tas2r106 | Tas2r138 |
|  | Tas2r124 | Tas2r125 | Saa2 | Calcr | Cxcl2 |
|  | Gnai1 | Cxcl12 | Sctr | Tas2r104 | Tas2r120 |
|  | Tas2r123 | Fpr-rs3 | Ptafr | Uts2d | Tas2r136 |
|  | Tas2r135 | Mtnr1a | Tas2r117 | Ghrl | Tas2r109 |
|  | Casr | Rgs14 | Tas2r102 | Ghrhr | Rxfp2 |
|  | Tas2r144 | Tas2r129 | Tas2r107 | Adcyap1 | Penk |
|  | P2ry14 | Agt | Fpr2 | Adm2 | Tas2r130 |
|  | Adcyap1r1 | Tas2r137 | Gpr17 | Cysltr1 | Tas2r139 |
|  | Ccr10 | F2r | Glp2r | Gpr18 | P2ry10 |
|  | Gpr4 | Prok1 | C5ar1 | Slc29a4 | Tas2r105 |
|  | Gnai2 | Gpr68 | Pik3r1 | Tas2r126 | P2ry4 |
|  | Qrfp | P2ry6 | Chrm3 | Ffar1 | Oxgr1 |
|  | F2rl3 | Pnoc | Niacr1 | Lpar4 | Tas2r118 |
|  | S1pr2 | Mtnr1b | Fpr1 | Ffar2 | Cxcr3 |
|  | Pthlh | P2ry12 | S1pr1 | Cxcr5 | S1pr4 |
|  | Tas2r119 | Cxcr1 | Gna15 | C3ar1 | Cxcl10 |
|  | Apln | P2ry13 | Pmch | Tas2r108 | Iapp |
|  | Gna11 | Chrm1 | Lpar6 | Hebp1 | Slc22a2 |
|  | Pth2 | Chrm4 | Cysltr2 | Fshr | Ccl5 |
|  | Ccr8 | Plcb4 | Grm1 | P2ry2 | Gpr44 |
|  | Avp | Vipr1 | Ido1 | Rgs10 | Adm |
|  | Calcb | Tas2r103 | Tas2r116 | Prok2 | Tacr1 |
|  | Cxcl1 | Cxcl3 | Ppbp | Cxcl5 | Nmu |
|  | Gnb1 | Uts2 | Gnat3 | Edn2 | Tacr3 |
|  | P2ry1 | Sucnr1 | Pik3ca | Edn3 | Hc |
|  | Xcl1 | Rgs18 | Cxcr2 | Pth2r | Nmb |
|  | Tac2 | Gcgr | Plcb3 | Gal | Adrbk1 |
|  | Gna14 | Anxa1 | Gnaq | Grp | C3 |
|  | Lhcgr | TECK | Cxcl13 | Npff | F2rl1 |
|  | F2rl2 | Edn1 | Gpr132 | Tshr | Tacr2 |
|  | Gprc6a | Arhgef25 | Ccl9 | Ccl6 | Cxcl16 |
|  | Ppy | Pyy | Vipr2 | Adora3 | Kiss1 |
|  | Trh | Pth1r | Adcy9 | App | Grm3 |
|  | Inmt | Grk5 | Rgs19 | Bdkrb2 | Grm6 |
|  | Gnai3 | Htr3b | Pparg | Kcnj3 | Sult1a1 |
|  | Ppp1r1b | Chrnb2 | Nos2 | Chrna4 | Chrna2 |
|  | Nos1 | Chrna3 | Chrna6 | Ndor1 | Mtrr |
|  | Tyw1 | Nos3 | Por | Chrne | Chrna5 |
|  | Chrna10 | Chrnd | Chrnb3 | Chrnb1 | Chrnb4 |
|  | Chrna7 | Chrna9 | Chrna1 | Chrng | Gpr119 |
|  | Nr3c1 | O3far1 | Gpr39 | Gpr62 | Rho |
|  | Kcnj6 | Opn5 | Slc6a2 | Slc6a3 | Kcnj4 |
|  | Kcnj12 | Kcnj9 | Kcnj2 | Kcnj5 | Gpr161 |
|  | Taar8c | Gpr45 | Gpr27 | Taar6 | Taar4 |
|  | Taar5 | Taar7b | Taar7d | Taar7e | Taar8b |
|  | Gpr88 | Gpr139 | Opn1sw | Gpr84 | Taar2 |
|  | Taar7a | Taar7f | Gpr21 | Gpr173 | Prlhr |
|  | Taar8a | Gpr3 | Gpr151 | Gpr135 | Gpr101 |
|  | Gpr6 | Gpr22 | Gpr61 | Gpr85 | Gpr37 |
|  | Ccbp2 | Gpr150 | Gpr19 | Gpr142 | Taar9 |
|  | Trhr2 | Gpr26 | Npy6r | Gpr63 | Gpr12 |
|  | Gpr176 | Taar3 | Gpr83 | Opn1mw | Pgr15l |
|  | Gpr165 | Brs3 | Rrh | Opn3 | Gpr37l1 |
|  | Rgr | Lsp1 | A3R | Calb2 | Cma1 |
|  | Nr2c2 | Esrrb | Nr3c2 | Nr1h4 | Thra |
|  | Esr2 | Nr2c1 | Nr2f1 | Rxra | Nr6a1 |
| 5-Methoxyindoleacetate | Asmt | Rxra | Rarg | Vdr | Rexo2 |
|  | Pparg | Nr6a1 | Nr0b2 | Thrb | Ikzf2 |
|  | Nr2c2 | Nr1h2 | Rxrb | Nr2e1 | Ikzf3 |
|  | Esrrb | Esr1 | Nr2e3 | Rxrg | Clnk |
|  | Nr3c2 | Rara | Nr2f2 | Nr1i3 | Mettl11b |
|  | Nr1h4 | Rarb | Esrrg | Nr2f6 | Ntmt1 |
|  | Thra | Pgr | Nr0b1 | Ppard | Ebf3 |
|  | Esr2 | Ppara | Esrra | Nr1h3 | Pcyt2 |
|  | Nr2c1 | Ar | Nr3c1 | Nudt5 | Tdo2 |
|  | Nr2f1 | Nr1h5 | Nr1i2 | Ikzf4 | Col2a1 |
|  | Plekha2 | Ddi1 | Olfr1261 | Plekha1 | Dnaja3 |
|  | Gm5422 | Psmd2 |  |  |  |
| 5-Hydroxyindoleacetic acid | Asmt | Akr1b3 | Ppard | Pcbd1 | Aifm3 |
|  | Aldh2 | Thra | Nr1h3 | Gast | Tac1 |
|  | Aldh3a2 | Esr2 | Tdo2 | Syp | Gal |
|  | Aox3l1 | Nr2c1 | Cbr1 | Gdnf | Tada3 |
|  | Aldh7a1 | Nr2f1 | Dhfr | Fev | Tmprss11d |
|  | Aox3 | Rxra | Htr1b | Hrh2 | Tmem176a |
|  | Aox4 | Nr6a1 | Drd4 | Prl | Mettl11b |
|  | Aldh1b1 | Nr1h2 | Qdpr | Crh | Ntmt1 |
|  | Aldh9a1 | Esr1 | Ddc | Oprm1 | Snca |
|  | Aox1 | Rara | Emc4 | Krt35 | Myc |
|  | Top1 | Rarb | Dst | Fip1l1 | Slc6a3 |
|  | Pparg | Pgr | Drd2 | Aco1 | Adh4 |
|  | Top1mt | Ppara | Hsd3b4 | Ireb2 | Gabra6 |
|  | Htr2c | Ar | Gm10681 | Kdm4c | Chat |
|  | Slc6a4 | Nr1h5 | Hsd3b5 | Aco2 | Adh5 |
|  | Htr2a | Rarg | Htr7 | Eno2 | Pth |
|  | Tph1 | Nr0b2 | Ndp | Gskip | Ypel3 |
|  | Htr1a | Rxrb | Il4i1 | Mccc1 | Polr3a |
|  | Spr | Nr2e3 | ENSMUSG00000069873 | Disc1 | Htr2b |
|  | Maob | Nr2f2 | Lao1 | Sst | Rbpsuh-rs3 |
|  | Comt | Esrrg | Drd1a | Pdpn | Olfr1143 |
|  | Tph2 | Nr0b1 | Cdca7l | Fdxr | Syngr4 |
|  | Nr3c1 | Esrra | Slc18a2 | Tomt | Rbpj |
|  | Gch1 | Nr1i2 | Maoa | Uroc1 | Fezf1 |
|  | Spr-ps1 | Vdr | Slc38a1 | Erp27 | Fezf2 |
|  | Mcee | Thrb | Bdnf | Prl3b1 | Htr3a |
|  | Nr2c2 | Nr2e1 | Drd3 | Synpr | Pde1a |
|  | Esrrb | Rxrg | Sh3rf3 | Men1 | Sh3rf2 |
|  | Nr3c2 | Nr1i3 | Sh3rf1 | Avpr1a | Elovl6 |
|  | Nr1h4 | Nr2f6 | Th | Adra1a | Prss21 |
| 1,3-Dimethyluracil | Themis | Themis2 | Themis3 | Lcp2 | Tppp |
|  | Acd | Tymp | Cfd | Dpcr1 | Six5 |
| L-Tyrosine methyl ester | Sult1a1 | Asnsd1 | Sulf1 | Sgsh | Fetub |
|  | Sult1d1 | Arsa | Sulf2 | Cpb2 | Rab8b |
|  | Urod | Arsk | Arsi | Ralgapb | Tph1 |
|  | Asns | Ids | Gns | Tomt | Ndufb9 |
|  | Th | Arsj | Arsg | Ddc | Pah |
|  | Unc80 | Arsb | Galns | Tat | Tph2 |
|  | Alb | Nalcn |  |  |  |
| 3-Methoxytyrosine | Yars2 | Yars | Qdpr | Ikbkap | Ddc |
|  | Omd | Tomt | Hdc | Srr | Htr2a |
|  | Pigv | Gsc2 | Maob | Htr2c | Pnpo |
|  | Aldh7a1 | Comt | Fech | Sds | Sdsl |
|  | Hsf5 | Snap29 | Gdf6 | Rgn |  |
| L-Pipecolic acid | Pipox | Orc2 | Clip2 | Slc2a13 | Snai1 |
|  | Il4 | Kif11 | Clip4 | Slc2a6 | Snai2 |
|  | Pin1 | Dctn1 | Clip3 | Slc2a12 | Snai3 |
|  | Atp5c1 | Slc3a2 | Agxt2l2 | Slc2a10 | Rev1 |
|  | Prep | Dpysl5 | Agxt2l1 | Slc2a8 | Gusb |
|  | Atp5a1 | Slc3a1 | Nudc | Umps | Ivd |
|  | Atp5b | Pcbp2 | Trit1 | H1foo | Acadl |
|  | Clpp | Pcbp1 | Gm1604A | Ky | Tubgcp3 |
|  | Phyh | Pcbp4 | Wfdc8 | Crym | AU018091 |
|  | Pin4 | Pcbp3 | Eppin | Slc11a1 | Gm21985 |
|  | Ccdc102a | Hspe1 | Spint5 | Slc11a2 | Slc12a2 |
|  | Abat | Ascl3 | Spint3 | Adat3 | Slc12a5 |
|  | Agxt2 | Wars | Wfdc6a | Adat2 | Slc7a2 |
|  | Wipf3 | Wars2 | Spint2 | Aass | Slc7a14 |
|  | Dao | Bysl | Wfdc6b | L3hypdh | Slc7a3 |
|  | Dmgdh | Slc30a6 | Tfpi2 | Rer1 | Slc7a4 |
|  | Ddo | Mcc | Ambp | Slc35d1 | Slc12a8 |
|  | Crmp1 | Slc30a5 | Spint1 | Slc35d2 | Slc7a1 |
|  | Dpysl4 | Slc30a7 | Tfpi | Slc35d3 | Slc12a9 |
|  | Dpysl3 | Nudt11 | Spint4 | Rps5 | Slc12a4 |
|  | Dpys | Nudt10 | Scg2 | Nit1 | Slc12a3 |
|  | Dpysl2 | Nudt7 | Cnot7 | Btd | Slc12a1 |
|  | Slc7a15 | Nudt1 | Cnot8 | Upb1 | Slc12a6 |
|  | Slc7a5 | Nudt2 | Rpl31 | Vnn1 | Slc12a7 |
|  | Slc7a12 | Nudt5 | Spag9 | Nadsyn1 | Txndc8 |
|  | Slc7a13 | Nudt8 | Aldh6a1 | Nit2 | Nme8 |
|  | Slc7a6 | Dcp2 | Spon1 | Vnn3 | Txndc2 |
|  | Slc7a9 | Nudt3 | Cetn3 | Slc30a9 | Mrps7 |
|  | Slc7a11 | Nudt4 | Gfod1 | Scrt1 | Txn1 |
|  | Slc7a8 | Prodh | Gfer | Rusc1 | Trdmt1 |
|  | Slc7a10 | Nudt14 | Gfod2 | Fdps | Dnmt1 |
|  | Slc7a7 | Clip1 | Dhdh | Scrt2 | Dgke |
| Creatine | creatine phosp. | Mlf1 | Acss1 | Hipk1 | Aldoc |
|  | MgADP | Murc | Cyb5r1 | Zhx1 | Aldoart1 |
|  | creatine | Nppa | Fn3k | Ucp3 | AC1L9JAC |
|  | Ckm | Xirp2 | Ip6k3 | Aldoa | Dctn1 |
|  | Slc25a4 | Popdc2 | Dctn3 | Pygl | Sypl2 |
|  | Vdac2 | Myo18b | Aox4 | Pygb | Gba |
|  | Mb | Actn3 | Atp5a1 | Ints6 | Hk1 |
|  | Vdac3-ps1 | Ldha | Rtn2 | Elovl7 | Hkdc1 |
|  | Vdac3 | Got1 | RFQ | Atp5f1 | Chrm2 |
|  | Vdac1 | Des | Asb10 | Terf2 | Gm17352 |
|  | Gamt | Zcchc9 | Atp2a2 | Suclg1 | Chrna1 |
|  | MgATP | Hspb7 | Aco2 | Pccb | non-guanine |
|  | Ckmt1 | Ak8 | Tpm4 | Ppp1r27 | Dld |
|  | hydrogen | Ampd1 | Actg2 | glutaryl | Tufm |
|  | N-phosphocreat. | Hspb3 | Lrrc39 | Asb9 | Nmt2 |
|  | A831275 | Gtf3c4 | Myl7 | N-methyl-3-pyr. | Ppip5k1 |
|  | Hrc | Pdk2 | Frmd5 | Dnajb14 | Ppip5k2 |
|  | atorvastatin | Myh8 | Adck3 | Fbxo33 | Nmt1 |
|  | dantrolene | Rbfox1 | Mtch1 | Rasgrf1 | Pgk1-rs7 |
|  | diltiazem | Got2 | Poll | Hadha | Lactb |
|  | isoproterenol | Lrrc2 | Samm50 | Prlr | Gm10175 |
|  | lovastatin | Acat1 | Tbca | Olfm2 | Oxct2b |
|  | glyceryl trini. | Casq1 | Parp4 | Slc6a8 | Atp5g2 |
|  | suxamethonium | Zfyve16 | Coq6 | Parg | Ube3b |
|  | Cmya5 | Usp13 | Slc2a4 | Cdk5r2 | Ube3c |
|  | Tcap | Hspa1l | Agxt2l1 | Smtnl1 | AGN-PC-088Y3L |
|  | Csrp3 | Nppb | Cct3 | 3,5-difluo.nza. | Pklr |
|  | Ttn | Ppp1r14c | Nme3 | Mib2 | 3-hydroxyb.yl-. |
|  | Myl3 | Ankrd1 | cefetamet | Kcns3 | 5-methylanthra. |
|  | Myoz2 | AC1NSA1Y | 5 tryptophan | Mib1 | Ankrd2 |
|  | Txlnb | Tpm2 | Gly-Gly-His | Slc36a2 | Bves |
|  | Cox6a2 | Pvalb | 2 tryptophan | Nme1 | Cs |
|  | Actn2 | Tmem38a | Oxct1 | Snx25 | Ap3b1 |
|  | Pgam2 | Fndc5 | Aldh4a1 | Slc25a12 | Glo1 |
|  | magnesium | Synpo2l | Ndufs2 | Fubp1 | Tpm3-rs7 |
|  | Eno3 | AV083437 | Aldob | Ispd | Tpm3 |
|  | Myom2 | Art1 | Hspe1 | Aox3 | fusidic acid |
|  | Myom1 | Mylk3 | Myh3 | Wdtc1 | Dnajc24 |
|  | Ldb3 | Tnni1 | Fsd2 | Pdha1 | Gabpa |
|  | Myl2 | Pfkm | Ncaph2 | Cdk14 | Tfam |
|  | Clpb | Cap2 | Tnnt1 | methyl guanidi. | Idh2 |
|  | Pygm | Oat | Rasgrf2 | pyridine-2,6-d. | Rps18 |
|  | Smpx | Cacna1s | Immt | TMPyP4 | Prdm10 |
|  | Myh2 | Xirp1 | C03725 | phospho-L-argi. | Asb12 |
|  | Myot | Cox7a1 | Grpel2 | Ndufb6 | 4btu |
|  | phosphate | Mybpc3 | Grpel1 | Gpx1 | Col15a1 |
|  | Myoz1 | Myadml2 | Hk2 | Dcaf6 | Hcls1 |
|  | Myl1 | Ppp1r3a | Sh3bgr | Nme2 | leptomycin |
|  | Trim54 | Hhatl | Slc35a3 | Ywhag | N,O-diacetylty. |
|  | Trdn | Hsph1 | Ndufs1 | Sarm1 | Raly |
|  | Ankrd23 | Strc | Gmfg | Got1l1 | Ndrg2 |
|  | Tnni3 | Catsper2 | Zc3hc1 | Denu | Glud1 |
|  | Tnni2 | Ak4 | Mkks | Immp1l | ketophenylbuta. |
|  | Myh4 | Hspa8 | Srpk3 | Gm20390 | Chi 11 |
|  | glycerol | Asb15 | Pgm2 | splitomicin | rigid one |
|  | nitrate | Kbtbd10 | Atp5b | Nme5 | anti-EpoR |
|  | sulphate | Ryr1 | Hspd1 | Fitm1 | ENSMUSG00000026809 |
|  | DB02490 | Alpk3 | Oxct2a | Mef2c | Mstn |
|  | Acta1 | Fundc2 | Kbtbd5 | 4-nitro-3-octa. | Dmd |
|  | Tnnt3 | Nme4 | Art5 | Gapdh | Scn5a |
|  | Nrap | Hspa1b | Stk38 | Cpt1b | Fxyd1 |
|  | Srl | Hspa9 | Ppp1r12b | reduced glutat. | 3-ethylben.ne-. |
|  | Cox8b | Asb3 | Acadm | hydrogen perox. | Cpt2 |
|  | Rpl3l | Asb14 | Ugp2 | xanthine | AB26213 |
|  | Lmod2 | Asb1 | Cct4 | calcium io.e A. | Nnt |
|  | Eef1a2 | Hspa1a | Tcp1 | berberine | Amd2 |
|  | Tmem182 | Nexn | Pikfyve | bisoprolol | Slc25a11 |
|  | acetate | Itgb1bp2 | Bbs10 | imipramine | Nfyb |
|  | calcium ions | Fabp3 | Cct2 | molsidomine | Fam168b |
|  | Myh6 | 5-Pyrrolidin-2. | Cct7 | N-ethylmaleimi. | Fam116a |
|  | Mylpf | AGN-PC-0JISI5 | Cct5 | streptomycin | Ikbkap |
|  | Atp2a1 | Nmrk2 | C7BzO | retinoic acid | Jph2 |
|  | Casq2 | Neb | Rxrg | acrylic acid | Ssbp2 |
|  | Tnnt2 | Etfdh | AGN-PC-0DAJZ4 | N,N'-methy.isa. | NPCC |
|  | Hfe2 | Myog | Cryab | mercury | 2,2,6,6-tetram. |
|  | Abra | Myf6 | Tmod1 | oleuropein | L-glucoside |
|  | Tnnc1 | Klhl31 | Psmg3 | peroxynitrite | 3-aminoben.cid. |
|  | Asb11 | Hspa12b | Cox6b1 | Irx4 | 4hu1 |
|  | Yipf7 | Hspa2 | Arl8b | compound 15e | Engase |
|  | Actc1 | Hspa4l | Pde4dip | Idh3a | Cox6b2 |
|  | Tpm1 | Hyou1 | alpha-amino-ep. | Gbas | Srpr |
|  | Obscn | Hspa12a | Sgcg | Pcca | Tenm1 |
|  | Apobec2 | Ankrd45 | Hk3 | C19-GAs | Hnrnpf |
|  | Asb8 | Hspa13 | succinyl-lysine | Oxr1 | Gm4862 |
|  | Myh7 | Hspa5 | adipyl | Etfa | Nme8 |
|  | Mypn | NST-1 | Slc25a29 | ENSMUSG00000097148 | Nme6 |
|  | Tmod4 | Hspa4 | Gart | Ndufb10 | Sirt2 |
|  | Sgca | Ryr2 | crotonyl-CoA | Uqcrc1 | Ldhc |
|  | Asb2 | Asb6 | crotonate | lactobionic ac. | Msrb1 |
|  | Cav3 | Ldhb | ENSMUSG00000019933 | Aldh2 | Msrb3 |
|  | Pln | Pdlim3 | glutaryl-CoA | Eif4g3 | Atp5g1 |
|  | Lmod3 | Hspb6 | Barhl2 | Gsta3 | Nell2 |
|  | Asb5 | Alpl | Klhl30 | Pfas | Aass |
|  | Ak1 | Pik3c2a | butyryl-CoA | Sln | Msrb2 |
|  | Adprhl1 | Trim63 | Uqcrc2 | xylotetraose | 2,4-difluoroan. |
|  | Myh1 | Mybpc1 | Aatk | Ank1 | diethanola. ol. |
|  | Mlip | Mdh2 | Dnahc8 | Atp5h | rutin |
|  | Tnnc2 | Echdc1 | Rps13-ps2 | Nme7 | Acly |
|  | Mybpc2 | Gpt2 | Rps13 | Ak5 | Sgpl1 |
|  | Dhrs7c | Gpt | cyclocreatine | 51Cr-EDTA | Fhl2 |

**Supplementary Table S2. Overlapping target proteins of the components of GS and differential metabolites.**

| **Overlapping target protein** | **Component of GS** | **Differential metabolite** |
| --- | --- | --- |
| ESR2 | 2’-Hydroxygenistein | Serotonin |
|  | Formononetin | 5-Hydroxyindoleacetic acid |
|  | Genistein | 5-Methoxyindoleacetate |
|  | Homoferreirin |  |
| ESR1 | 2’-Hydroxygenistein | 5-Hydroxyindoleacetic acid |
|  | Apigenin | 5-Methoxyindoleacetate |
|  | Aromadendrin |  |
|  | Chrysoeriol |  |
|  | Formononetin |  |
|  | Genistein |  |
|  | Homoferreirin |  |
|  | Luteolin |  |
| PGR | Apigenin | 5-Hydroxyindoleacetic acid |
|  | Aromadendrin | 5-Methoxyindoleacetate |
|  | Luteolin |  |
| AR | Chrysoeriol | 5-Hydroxyindoleacetic acid |
|  | Luteolin | 5-Methoxyindoleacetate |
| ALB | Formononetin | L-Tyrosine methyl ester |
| GBA | Isovitexin | L-Fucose |
|  | Schaftoside | Creatine |
|  | Vanillic acid |  |
|  | Vicenin-2 |  |
| MAOB | β-sitosterol | Serotonin |
|  |  | 5-Hydroxyindoleacetic acid |
|  |  | 3-Methoxytyrosine |
| CMA1 | Isovitexin | Serotonin |
|  | Vicenin-2 |  |
|  | Vicenin-3 |  |
| THRB | β-sitosterol | 5-Hydroxyindoleacetic acid |
|  |  | 5-Methoxyindoleacetate |

The MS and MS/MS spectral data of schaftoside.

The MS and MS/MS spectral data of luteolin.

The MS and MS/MS spectral data of apigenin.

**Supplementary Figure S1.** The MS and MS/MS spectral data of major constituents of GS, including schaftoside, luteolin and apigenin.


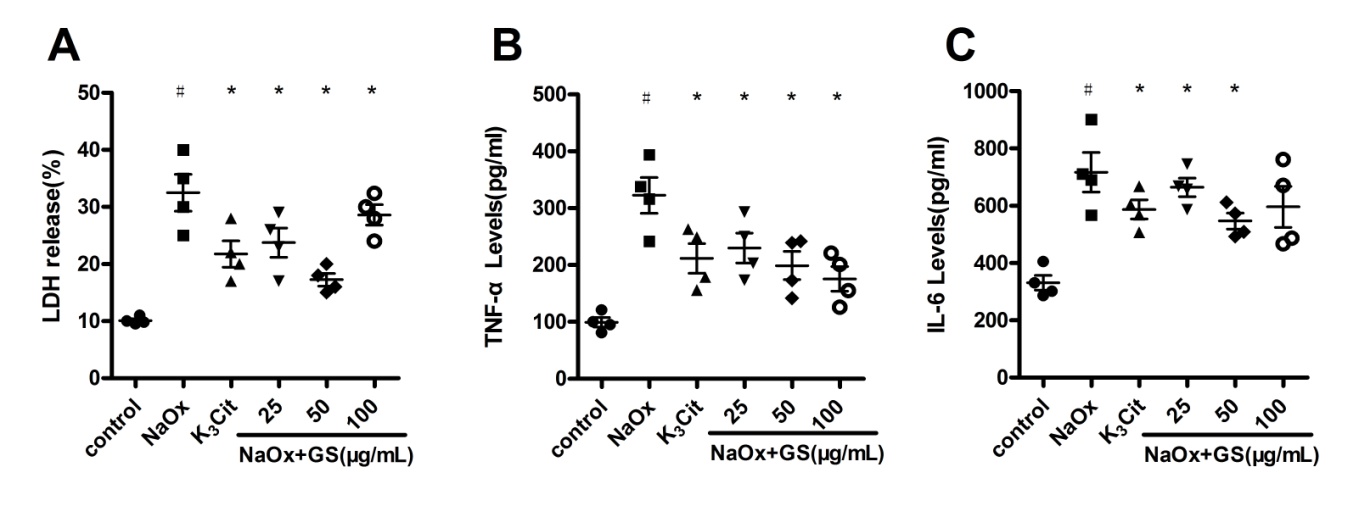


**Supplementary Figure S2.** The effects of GS extraction on oxalate-stimulated HK-2 cells. (A) LDH release detection. (B) The secretion of TNF-α. (C)The secretion of IL-6. ^#^P < 0.05 vs. untreated cells; *P < 0.05 vs. cells treated with NaOx alone.


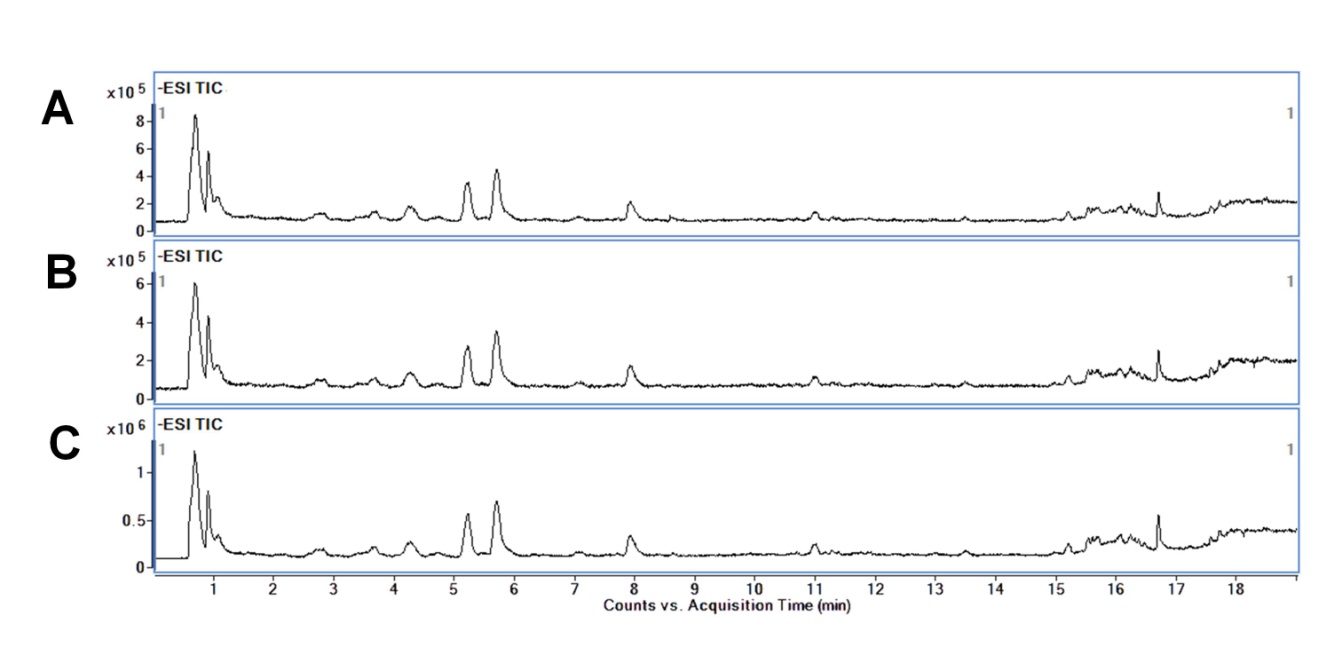


**Supplementary Figure S3.** Representative total ion chromatogram (TIC) obtained for the ESI-negative ion in the control group (A), oxalate group (B) and oxalate + high dose GS group (C).


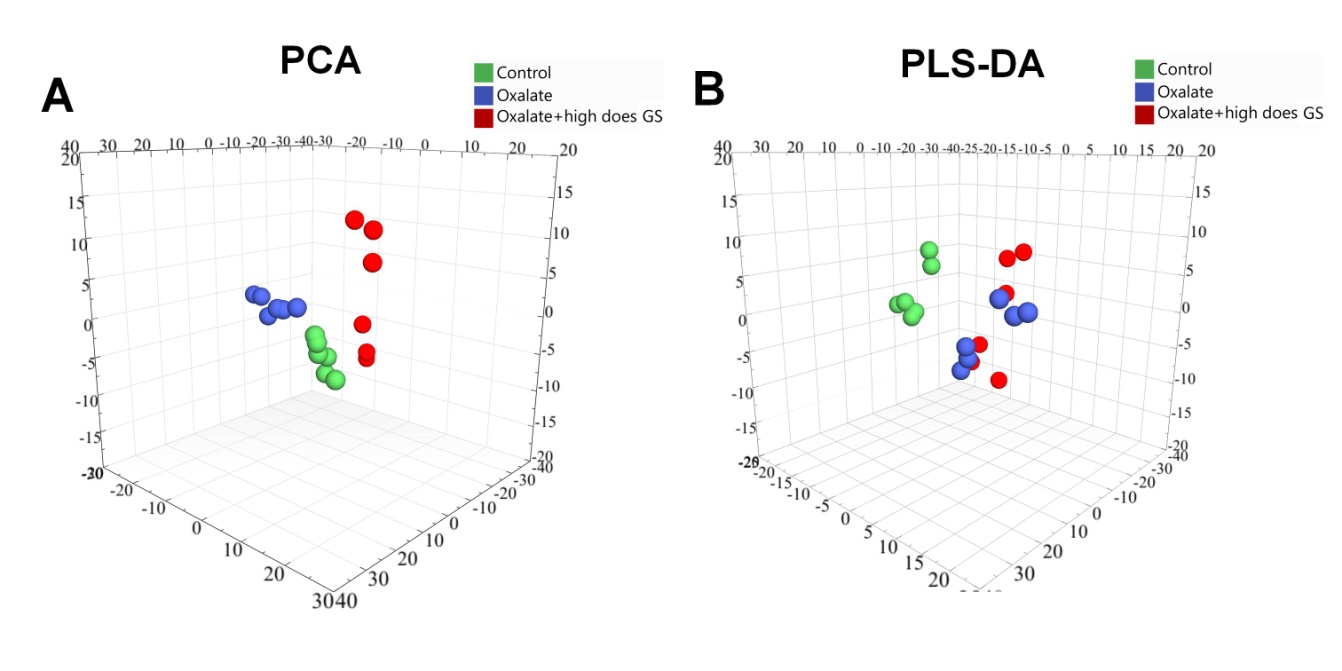


**Supplementary Figure S4.** Score plots of PCA and PLS-DA in ESI negative ion mode. (A) PCA score plots based on the urine metabolic profiling of the control group, oxalate group and oxalate + high dose GS group. (B) PLS-DA score plots of urine samples obtained from the control group, oxalate group and oxalate + high dose GS group.


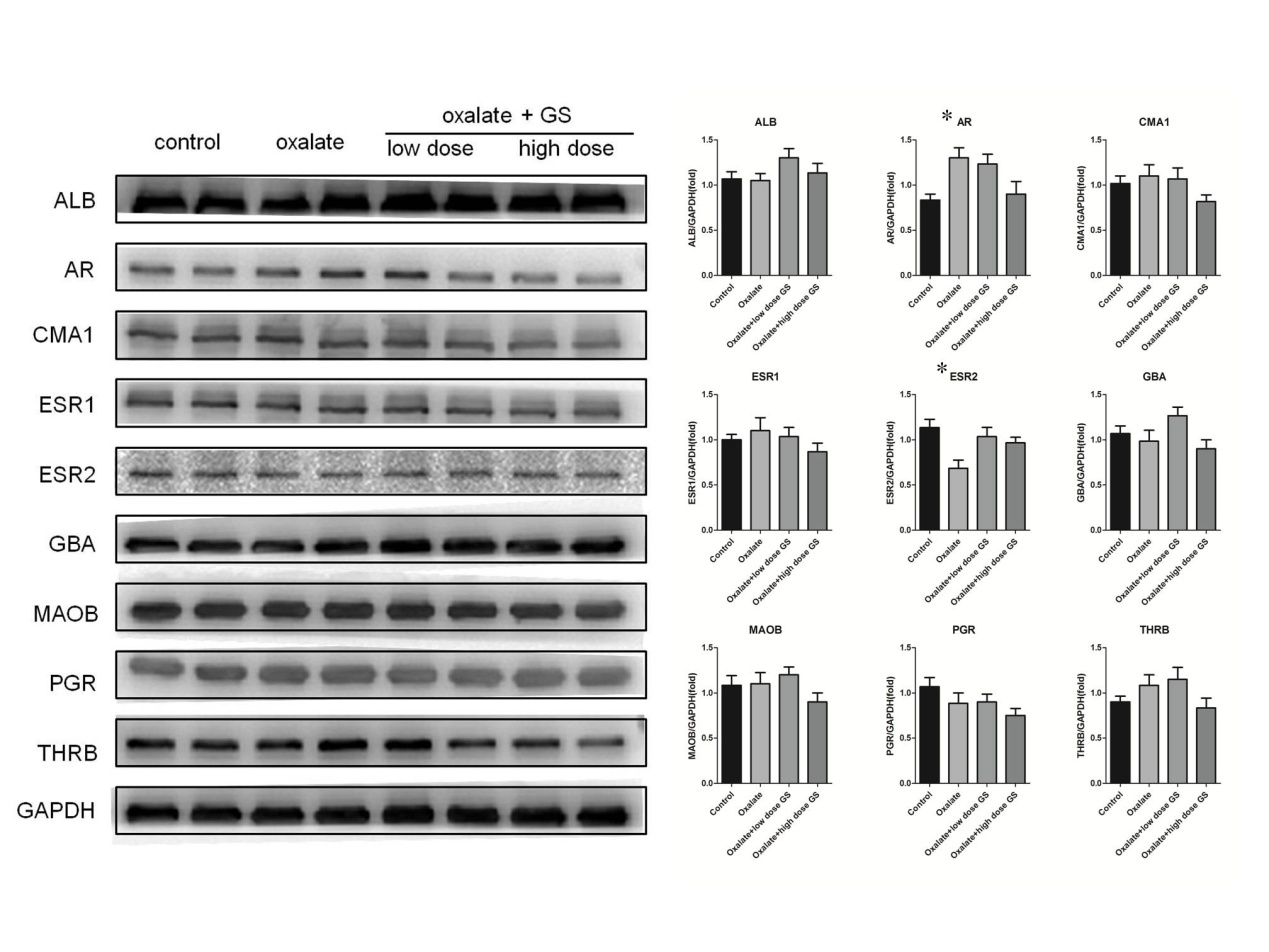


**Supplementary Figure S5.** Effects of GS on the expression of ALB, AR, CMA1, ESR1, ESR2, GBA, MAOB, PGR and THRB in mice. The western blotting results of ALB, AR , CMA1, ESR1, ESR2, GBA, MAOB, PGR and THRB in the corticomedullary region of kidney. Statistical comparisons were performed using a Kruskal-Wallis H test (AR, ESR2:*p < 0.05, ALB, CMA1, ESR1, GBA, MAOB, PGR and THRB: p > 0.05 )
